# Are physical activity referral scheme components associated with increased physical activity, scheme uptake, and adherence rate? A meta-analysis and meta-regression

**DOI:** 10.1186/s12966-024-01623-5

**Published:** 2024-08-02

**Authors:** Eriselda Mino, Klaus Pfeifer, Coral L. Hanson, Michael Schuler, Anna Brandmeier, Sarah Klamroth, Inga Naber, Anja Weissenfels, Sheona McHale, Karim Abu-Omar, Peter Gelius, Stephen Whiting, Kremlin Wickramasinghe, Gauden Galea, Wolfgang Geidl

**Affiliations:** 1 Department of Sport Science and Sport, Friedrich-Alexander-Universität Erlangen-Nürnberg (FAU), Gebbertstraße 123b, Erlangen, 91058 Germany; 2 School of Health and Social Care, Edinburgh Napier University, Sighthill Campus, Edinburgh, EH11 4DN UK; 3 Institute of Clinical Epidemiology and Biometry, University of Würzburg, Josef-Schneider-Str. 2/ D7, Würzburg, 97080 Germany; 4 Department of Applied Health Sciences, University of Applied Sciences, Bochum, Germany; 5 Institute of Sport Sciences, Université de Lausanne, Lausanne, Switzerland; 6 Special Initiative for Noncommunicable Diseases and Innovation (SNI), WHO Regional Office for Europe, Copenhagen, Denmark

**Keywords:** Physical activity, Physical activity referral scheme, Exercise referral scheme, Physical activity prescription, Exercise prescription, Referral and consultation

## Abstract

**Background:**

Physical activity referral schemes (PARS) are composed of various components, such as a written prescription or a person-centered approach. The role of these components in their effectiveness is yet to be understood. Therefore, we aimed to explore the relationships between PARS components and physical activity, scheme uptake, and adherence rate; and to estimate the effect of PARS.

**Methods:**

We searched Scopus, PubMed, Web of Science, CINAHL, ScienceDirect, SpringerLink, HTA, Wiley Online Library, SAGE Journals, Taylor & Francis, Google Scholar, OpenGrey, and CORE. Eligible studies were published between 1990 and November 2023 in English or German, investigated PARS with participants aged ≥ 16 years, and reported physical activity, scheme uptake, or scheme adherence. Separate random-effects meta-analysis by comparison group were conducted for physical activity. Scheme uptake and adherence rates were pooled using proportional meta-analysis. The components were analyzed via univariate meta-regression. We rated the risk of bias using RoB2 and ROBINS-I, and the certainty of evidence using GRADE.

**Results:**

Fifty-two studies were included. PARS were more effective in increasing physical activity than usual care (k = 11, *n* = 5046, Hedges’ g = 0.18, 95%CI 0.12 to 0.25; high certainty of evidence). When PARS were compared with physical activity advice or enhanced scheme versions, the pooled Hedges’ g values for physical activity were -0.06 (k = 5, *n* = 1082, 95%CI -0.21 to 0.10; low certainty of evidence), and 0.07 (k = 9, *n* = 2647, 95%CI -0.03 to 0.18; low certainty of evidence) respectively. Scheme uptake was 87% (95%CI 77% to 94%, k = 14, *n* = 5000) across experimental studies and 68% (95%CI 51% to 83%, k = 14, *n* = 25,048) across non-experimental studies. Pooled scheme adherence was 68% (95%CI 55% to 80%, k = 16, *n* = 3939) and 53% (95%CI 42% to 63%, k = 18, *n* = 14,605). The meta-regression did not detect any significant relationships between components and physical activity or scheme uptake. A person-centered approach, screening, and brief advice were positively associated with scheme adherence, while physical activity sessions were negatively associated.

**Conclusion:**

PARS are more effective in increasing physical activity than usual care only. We did not identify any components as significant predictors of physical activity and scheme uptake. Four components predicted scheme adherence, indicating that the component-effectiveness relationship warrants further research.

**Supplementary Information:**

The online version contains supplementary material available at 10.1186/s12966-024-01623-5.

## Background

The promotion of physical activity (PA) by healthcare professionals has been proposed as a paramount strategy to foster an active society [1]. Physical activity referral schemes (PARS) are a promising intervention that allow healthcare professionals to advocate for PA and integrate its promotion into routine care. Previous seminal evidence syntheses have pointed to favorable but small effects of PARS on PA, and to considerable variation in design and implementation [2–4]. In 2014, the National Institute for Health and Care Excellence (NICE) in the United Kingdom (UK) recommended that future research should focus on increasing understanding of what influences effectiveness and cost effectiveness of PARS [5]. Since then, PARS research has mainly focused on participant-level factors (e.g., age, gender and socio-economic status of referrals), system-level factors (e.g., financial reimbursement), and scheme characteristics (e.g., setting, duration and intensity, costs) [6–9]. However, little attention has been paid to the role of the components that contribute to PARS complexity and heterogeneity. PARS content varies greatly as it can be grounded on various theories or approaches, such as a person-centered approach, and made up of many other standalone interventions, such as brief advice or PA sessions [10]. These separate and potentially active parts of PARS content are referred to as scheme components.

PARS diversity and intricacy are the result of over 30 years of organic development in different countries and healthcare systems. The number of schemes is increasing due to their potential to change PA behavior. For example, the number of European Union member states reporting a national program of healthcare-based PA counseling or prescription increased from approximately 46% to 79% between 2015 and 2018 [11]. Underlying healthcare systems are complex and heterogeneous [12], contributing to the well acknowledged variety and complexity of PARS interventions [13, 14]. Within this complexity, PARS consist of combinations of behavioral support activities (brief advice, counseling session(s), PA sessions) and guiding principles (person-centered approach, individualized content) [10]. These individual components are assumed to contribute to the effectiveness of PARS in varying degrees. Schemes containing the core components of the Swedish model (i.e., patient-centered approach, individually tailored PA recommendations, written prescription, and structured follow-up) have been deemed effective, although it is currently unclear which components are more likely to result in increased PA [15]. In addition, previous research underscores the need to explore the factors that lead to optimal program uptake and adherence, which are necessary to demonstrate the true impact of PARS [9, 16, 17]. What is lacking is a proper understanding of the component-effectiveness relationship [16].

A better understanding of how components may shape scheme effectiveness can help program developers to design PARS that are only as complex as needed [18] or modify existing PARS to increase their effectiveness. Identification of the most effective core components could result in a focus on PARS optimization, more cost-efficient schemes, and improvements in participant outcomes. We have previously identified 19 components [10] and in this study we aimed to examine their effect on PA outcomes, scheme uptake and adherence rates.

## Methods

We analyzed the overall effect of 19 PARS components through meta-analysis and then used univariate meta-regression to examine the impact of each component. This analysis builds upon our systematic review [10], which followed the PRISMA guidelines [19], and the review protocol [20].

### Literature sources and inclusion criteria

The literature search was performed in Scopus, PubMed, Web of Science, CINAHL, ScienceDirect, SpringerLink, HTA, Wiley Online Library, SAGE Journals, Taylor & Francis, Google Scholar, OpenGrey, and CORE. The time searched in the previously published systematic review was from 1990 to January 2023 [10]. We updated the search in November 2023 (Additional file 1). Two independent reviewers (EM, AB) screened the articles identified from the updated search against the eligibility criteria. Experimental, quasi-experimental, and observational studies published in English or German were included in the systematic review if:

- **P**opulation: The participants were aged ≥ 16 years.
- **I**ntervention: The study evaluated any intervention labeled as PARS, exercise referral schemes, or exercise on prescription or any similar intervention, such as PA counseling that included at least some form of documentation, such as a prescription or referral form.
- **C**omparison: The PARS was compared to usual care, PA advice, alternative intervention (scheme versions), or no intervention. When the PARS was compared with PA advice, the comparison group received only advice about PA from the healthcare professional and no further intervention. Some studies compared standard PARS with enhanced versions, typically extending beyond of the standard scheme by incorporating additional components or increasing session frequency. For example, the standard version included a written prescription and counseling support sessions, whereas the enhanced version integrated additional PA sessions.
- **O**utcomes: The study reported either PA level, scheme uptake, or adherence rates.
- **S**etting: The PARS (or referral to the PARS) was initiated in primary or secondary healthcare, as noted in the included study. Primary healthcare generally includes a general practitioner or practice nurse, and secondary healthcare includes more specialized care, such as a diabetologist, cardiologist, or mental health practitioner.

### Risk of bias

We used the Cochrane risk-of-bias tool for randomized trials (RoB2) [21] to assess risk of bias for experimental studies, and Risk of Bias in Non-randomized Studies-of Interventions (ROBINS-I) [22] for quasi-experimental and observational studies. Two authors (EM and AB) assessed studies independently and resolved any disagreements through discussion until consensus was reached. We used the RoB VISualisation (robvis) to create risk of bias graphs in R [23].

### Quality of evidence

We used the Grading of Recommendations Assessment, Development and Evaluation (GRADE) approach to assess the quality of evidence at outcome level [24]. One author (EM) rated the quality of evidence as very low, low, moderate, or high. For randomized trials contributing to the meta-analysis of PA outcome, the rating started at high quality of evidence. For the uptake and adherence rate meta-analysis the quality rating started as low. We downgraded the quality of evidence if serious or very serious limitations were present in domains of risk of bias, imprecision, inconsistency, indirectness, and publication bias. We present results as GRADE evidence profiles and summary of findings tables [25].

### The PARS components

The 19 components investigated in this paper (Table 1) were previously identified by our team through a content analysis of various PARS models [10].

**Table 1:** PARS components

Person-centered approach	The use of a patient-/person-centered approach to delivering PARS by taking account of participants’ unique characteristics, needs, past history, and preferences with the aim of creating *shared decision-making between the healthcare/exercise provider and the PARS participant, and a respectful, empowering environment* that results in changed PA behavior.
Individualized content	Tailoring/selecting *appropriate PARS intervention content* to match participants’ unique characteristics, heath status, needs, past history, and preferences that results in changed PA behavior.
Behavior change theory	Employing a behavior change theory as the theoretical foundation of the PARS.
Behavior change techniques	Using at least one behavior change technique at some point during the PARS.
Screening	Systematically assessing an individual’s eligibility for the PARS (not for the study).
Brief advice	Targeted purposeful conversation about the topic of PA between the healthcare professional and the PARS participant (up to 10 min).
Written prescription	A written formal document (one to two pages) that contains specific instructions or recommendations on PA for the participant.
Written materials	Handing out materials that target the behavior change of the participant.
Referral	A written formal document (one to two pages) that serves as a communication/transfer tool of the participant to healthcare/PA professionals or programs.
Baseline consultation	A structured consultation session (30–60 min) at the very beginning of the PARS.
Exit consultation	A re-visit consultation (30–60 min) at the end of the scheme.
Counseling support session(s)	One or more structured counseling sessions (30–60 min) that guides the participant in the realization of PA behavior change efforts.
PA sessions	PA activities that are an integral part of the scheme.
Education session(s)	One or more structured sessions aimed at providing information relevant for enhancing PA behavior.
Action for non-attendance	Any action taken to address participants’ lack of attendance with the aim of increasing further engagement with the scheme.
Structured follow-up	Systematic and scheduled interactions (5–20 min) with the participants aimed at progress monitoring and ongoing support.
PA network	Interconnected group of healthcare professionals, PA professionals and/or PA opportunities that are available to the PARS participants to ensure support for behavior change and/or continuation after scheme completion.
Feedback to referrer	Participants’ progress report to the referring healthcare professional at the scheme completion.
Exit strategies/routes	The use of strategies to encourage behavior change continuation after scheme completion.

For a more thorough description of the components see Additional file 3 in [10] (page 19)

### Outcome data extraction

Quantitative data were extracted by one reviewer (EM) using Microsoft Excel spreadsheets. We extracted data related to PA outcome, PARS uptake and adherence rates, as well as study-level characteristics. For the PA outcome, we extracted sample size, reported effect size (ES) if available, and mean and standard deviation (SD) at baseline and follow-ups. Otherwise, we extracted other available statistics that would enable an ES calculation. We contacted authors of 13 primary studies where insufficient data were reported, but only two supplied the requested data. We focused on total PA but when not available, we extracted other reported PA outcomes such as moderate to vigorous PA or walking time. For uptake, the total number of persons offered the PARS and the number entering the scheme were extracted. For adherence, the number who took up and adhered to the PARS were extracted.

### Data synthesis

#### Effect size calculation

The summary statistics and PA instruments differed across studies, thus we used the standardized mean difference Hedges’ g [26, 27] as a uniform measure of effect, using the Cohens’ d interpretation as small (g = 0.2), medium (g = 0.5), and large (g = 0.8) [28]. For independent group comparisons we used the mean difference between groups and the pooled SD at post-scheme. For dependent samples we subtracted pre- from post-scheme mean value and divided by baseline SD. For studies that reported only standard error (SE), we multiplied SE by the square root of the sample size to obtain SD. When only range was reported, in absence of other similar studies to borrow a SD, we adopted a solution proposed by Walter and Yao [29] and used a correction factor f and the sample size. When median and quartile range were reported we followed the formulas from Wan et al. [30]. If only the 95% confidence intervals (CI) were presented, the difference was divided by 3.92 and multiplied with the sample size square root to obtain SD [21]. In the case of dichotomous PA outcomes we transformed the reported odds ratios to Hedges’ g [26]. All the summary statistics transformations were done in Microsoft Excel (Additional file 2).

#### Meta-analysis

PA data were pooled using separate random-effects meta-analysis by comparison group (usual care, PA advice, and enhanced PARS). Only experimental studies with sufficient data to calculate ES were combined, given that they offer better evidence than other types of studies. We also pooled pre-post studies separately to experimental studies. The first available follow-up post-scheme was pooled. This is because it was the most consistently reported follow-up, mostly ranging from post-scheme to three months. The few cases reporting only six and nine month outcomes were subjected to sensitivity analysis and retained in the analysis if robustness was not compromised. Some studies measured the PA outcome using more than one instrument. As most instruments were self-report, we included self-reported outcomes as a first preferred option in the analysis. In the few studies where this was not available, we included objective measures. We used the DerSimonian-Laird estimator to adjust the weight for each study according to the heterogeneity variance (tau-squared, τ^2^) [31]. Additionally, the Knapp-Hartung adjustment was applied to CIs of the pooled ES. The results are presented as standardized mean differences (Hedges’ g) and 95% CI. To make the results more tangible for the clinicians and policy makers, we calculated the number needed to treat (NNT) from Hedges’ g using the Kraemer and Kupfer 2006 method [32].

To test the robustness of the pooled ES we searched for outliers and influential cases based on the leave-one-out method [33]. Statistical heterogeneity was investigated using I^2^ [34] (where 25% low; 50% moderate, 75% substantial). Additionally, we added prediction intervals [35] to the forest plot to show the expected true effects for 95% of similar future studies. We used contour-enhanced funnel plots of Hedges’ g against SE to visually explore publication bias, and in case of more than 10 studies per meta-analysis, we conducted the Egger’s regression test for small-study effects [36].

Data on uptake and adherence rate were pooled using a proportional meta-analysis with the aim of presenting a descriptive analysis of how participants engage with PARS rather than assessing effect. The data were first transformed using Freeman-Tukey double arcsine transformation and back transformed using the inverse logit transformation [37]. The Wilson-Score interval method is used to estimate the 95% CI. We did not assess publication bias for the uptake and adherence meta-analyses given that it is not suggested in these types of data [37].

#### Sub-group analysis

In case of low heterogeneity, no further sub-group analysis was made. Subgroup analysis was conducted assuming a common estimate of between-study heterogeneity between subgroups [26]. The potential explanatory characteristics were pre-specified in the review protocol: geographical location, study design, risk of bias, follow-up, population characteristics, and scheme length [20].

#### Meta-regression

We used univariate meta-regression with a categorical predictor to investigate whether PA, uptake, and adherence rates (as measures of effectiveness) were associated with the presence of specific PARS components (Table 1). Meta-regression was performed only for the components for which 10 or more studies were available (at least five having the component, five not). Components that were not associated with the outcome measure, were excluded (e.g., exit consultations were excluded from components associated with uptake). We conducted additional post-hoc meta-regression analysis using the total number of components as the predictor variable.

All the analysis were done using R studio software (4.3.0) [33, 38]. The analysis scripts are available via R markdown in Open Science Framework (https://osf.io/dv8fb/?view_only=1703f57bd7f74c6ca0786e7093b531ec).

## Results

### Study selection

**Fig. 1:**
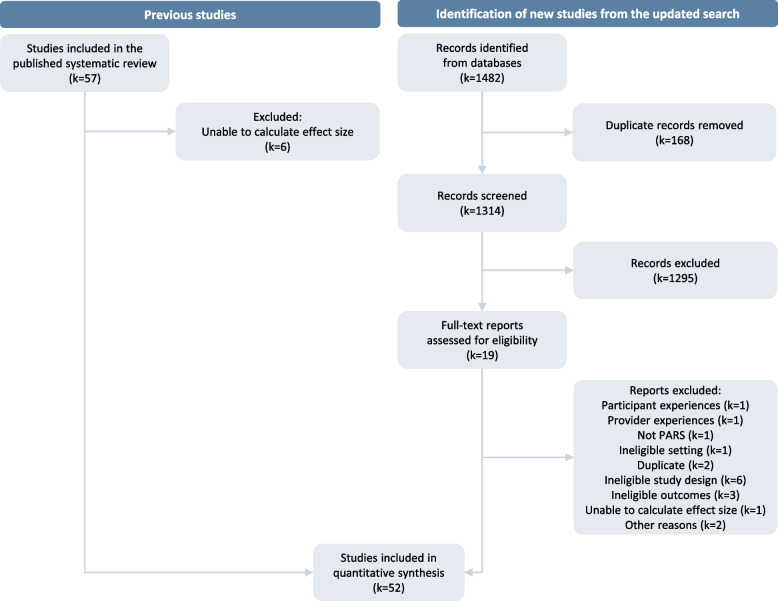
Study selection flow diagram. *k* number of studies, *for a more detailed description of the flow of studies through the systematic review please see figure 1 in [10]

From 57 studies included in our first systematic review [10], six were excluded because of insufficient data to compute ES (Additional file 3). One study was identified from the updated search [39]. In total, 52 studies [39–90] were included in the analysis (Fig. 1).

### Study characteristics

The included studies were experimental (k = 28, *n* = 9730) and non-experimental (k = 24, *n* = 28,405). The RCTs compared PARS with usual care (k = 11), PA advice (k = 5), and enhanced scheme versions (k = 9). Most studies (k = 50) targeted those with or at risk of non-communicable diseases (physical inactivity in combination with other risk factors such as overweight/obesity, elevated blood pressure, history of myocardial infarction, hypercholesterolemia, impaired glucose tolerance, smoking). PARS length ranged from one day (one-time intervention) to two years. Most studies (65%) were conducted in Europe, and follow-up time ranged from scheme completion to 15 months (Table 2). Of the 41 studies reporting PA, four measured PA via accelerometers or pedometers, 30 via questionnaires, and seven via both methods (Additional file 4). Uptake and adherence were measured via self-report or attendance records. Definitions of uptake and adherence differed slightly between studies (Additional file 5). Two broad categories of PARS were identified; those based on a written prescription (prescription scheme) and those with a referral to another healthcare/PA professional that might include additional prescription (referral scheme). Typically, for prescription schemes, uptake was defined as attendance at the first scheme activity, such as the initial counseling support session. The level of participation in counseling support sessions or other scheme activities was used as a measure of adherence. For referral schemes, uptake was usually defined as attending at least a baseline consultation and/or one PA session. Attendance at PA sessions was the most common measure of adherence.

**Table 2:** Characteristics of the included studies

**Study**	**Country**	**Study design**	**Participants**	**N**	**Female**	**Age** **(mean±SD)**	**PARS** **(length)**	**Comparison**	**Follow-up** (months)*	**PA**	**Uptake**	**Adherence**
Aittasalo et al. 2006 [40]	Finland	RCT	NCDs or at risk	203	154	20-65 (47±11)	One-time	Usual care	0	x	-	-
Andersen et al. 2020 [41]	Sweden	Observational follow up	NCDs	355†/400	276	18-90 (62±14)	1 year	Standard PARS	12	x	x	-
Bellanger et al. 2023 [39]	France	RCT	NCDs or at risk	121	55	IG: 59±8 CG: 60±9	12 months	Advice	12	x	-	-
Bredahl et al. 2011 [42]	Denmark	Quasi-experimental	NCDs or at risk	125†/337	231	54±12	10 months	-	0	x	-	-
Buckley et al. 2020 [43]	UK	Quasi-experimental	NCDs	52† /68	25	IG: 57±12 CG: 53±16	18 weeks	-	2	x	x	x
Crone et al. 2008 [44]	UK	Observational study	Physical or mental health problems	2901	1636	51±14 42±14	8-12 weeks	-	0	-	x	x
Dinan et al. 2006 [45]	UK	Prospective cohort study	NCDs or at risk	242	-	-	8 weeks	-	-	-	x	x
Dodd-Reynolds et al. 2020 [46]	UK	Embedded mixed-methods	Overweight, obese	950† /3600	2487	51±15	24 weeks	-	0	x	x	x
Duda et al. 2014 [47]	UK	Cluster RCT	NCDs	347	253	<30-65+	3 months	Standard PARS	0	x	-	-
Edmunds et al. 2007 [48]	UK	Observational study	Overweight, obese	49	41	44±14	12 weeks	-	0	-	-	x
Elley et al. 2003 [49]	New Zealand	Cluster RCT	CHD/CVD risk	878	582	40-79 (IG: 57± 10) (CG: 58±11)	3 months	Usual care	9	x	x	x
Foley et al. 2011 [50]	New Zealand	Comparative analysis	NCDs or at risk	5441	-	59±14	3-4 months	-	0	-	x	x
Fortier et al. 2011 [51]	Canada	RCT	NCDs	120	83	18-69 (47±11)	13 weeks	Standard PARS	0	x	x	x
Gademan et al. 2012 [52]	Netherlands	Non-RCT	Multi-ethnic disadvantaged	121† /514	-	IG: 45±10 CG: 41±12	6 months	-	0	x	x	x
Galaviz et al. 2013 [53]	Canada	Pre-post with controls	Obese	12† /35	12†	25-45	8 weeks	-	0	x	-	-
Gallegos-Carrillo et al. 2017 [54]	Mexico	Cluster RCT	Mild hypertension > 5 years	177	163	35-70 (IG: 50±1) (CG: 51±0)	16 weeks	Advice	2	x	x	x
Hanson et al. 2013 [55]	UK	Observational cohort study	NCDs	638† /2233	1327	16-75 (53±15)	24 weeks	-	0	x	x	x
Hanson et al. 2021 [56]	UK	Longitudinal mixed-methods	NCDs	92† /136	65	<50-70+	PARS	-	3	x	x	x
Harrison et al. 2005a [57]	UK	RCT	CHD risk	330† /545	363	18-60+	12 weeks	Standard PARS	3	x	-	-
Harrison et al. 2005b [58]	UK	Prospective register-based	NCDs or at risk	6610	4016	51±12	12 weeks	-	-	-	x	-
Hesketh et al. 2021 [59]	UK	Pragmatic trial	NCDs	67† /154	27	48±11	12 weeks	-	0	-	x	x
Isaacs et al. 2007 [60]	UK	RCT	CVD risk	606	421	40-74 (57±8)	10 weeks	Advice	4	x	x	x
James et al. 2017 [61]	Australia	Pragmatic RCT	NCDs	203	143	20-85 (57±13)	13 weeks	Usual care	0	x	x	x
Kallings et al. 2009a [62]	Sweden	uncontrolled clinical trial	NCDs or at risk	240	180	51±13	One-time	-	6	-	-	x
Kallings et al. 2009b [63]	Sweden	RCT	Overweight, abdominal obesity	101	43	67-68	One-time	Usual care	6	x	-	-
Kolt et al. 2012 [64]	New Zealand	RCT	NCDs or at risk	278	178	≤65 (73+6)	3 months	Standard PARS	0	x	-	x
Lawton et al. 2008 [65]	New Zealand	RCT	NCDs	1089	1089	40-79 (58±7)	9 months	Usual care	3	x	x	x
Leijon et al. 2010 [66]	Sweden	Prospective cohort study	NCDs or at risk	2612† /3300	1740	54±14	PARS	-	3	-	-	x
Livingston et al. 2015 [67]	Australia	Cluster RCT	prostate cancer	147	0	39-84 (65±8)	12 weeks	Usual care	0	x	-	x
Lord et al. 1995 [68]	UK	Pre-post	CVD risk	419	198	18-65	10 weeks	-	-	-	x	x
Lundqvist et al. 2020 [69]	Sweden	RCT	Metabolic risk factors	190	94	27-77 (57±10)	2 years	Standard PARS	0	x	-	x
Martín-Borràs et al. 2018 [70]	Spain	RCT	NCDs	422	257	18-85 (IG: 69±8) (CG: 68±8)	3 months	Usual care	3	x	-	x
Morén et al. 2016 [71]	Sweden	RCT	Acute TIA	60	47	49-90 (IG: 69±9) (CG: 72±8)	6 months	Usual care	0	x	-	-
Murphy et al. 2012 [72]	UK	Pragmatic RCT	NCDs	1795	1415	16-88 (52±14)	12 months	Usual care	0	x	x	x
Pardo et al. 2014 [73]	Spain	Longitudinal study	NCDs	242† /323	239	45-80 (62±8)	6 months	-	0	x	-	x
Petrella et al. 2010 [74]	Canada	Cluster RCT	Healthy community dwelling adults	329	206	55-85 (64±7)	PARS	Standard PARS	0	x	-	-
Pfeiffer et al. 2001 [75]	USA	RCT	NCDs	49	44	62-92 (74±1)	PARS	Advice	1.5	x	-	-
Prior et al. 2019 [76]	UK	Longitudinal study	NCDs or at risk	271	153	57±14	6 months	-	0	x	x	x
Riera-Sampol et al. 2020 [77]	Spain	RCT	CVD risk	263	153	35-75 (62±8)	12 months	Standard PARS‡	0	x	-	-
Romé et al. 2009 [78]	Sweden	RCT	NCDs	245† /525	168†	20-80 (IG: 55±12, CG: 54±13)	4 months	Standard PARS	0	x	x	x
Samdal et al. 2019 [79]	Norway	Pragmatic RCT	NCDs or at risk	81† /118	91	48±13	3 months	Usual care	3	x	-	-
Sjöling et al. 2011 [80]	Sweden	Pre-post with repeated measures	Mild to moderate hypertension	31	11	43-71 (61±7)	15 months	-	0	x	-	-
Sørensen et al. 2008 [81]	Denmark	RCT	NCDs	42	31	53 (95%CI: 49-57)	10 months	Standard PARS	0	x	x	x
Sørensen et al. 2011 [82]	Denmark	Observational follow up study	NCDs or at risk	449	264	57±11	10 months	-	0	x	-	-
Stewart et al. 2017 [83]	UK	Longitudinal repeated measures	NCDs	226† /407	243	16-75+	12 weeks	-	0	x	x	x
Swinburn et al. 1998 [84]	New Zealand	RCT	NCDs	456	281	49±15	PARS	Advice	1.5	x	-	-
Taylor et al. 1998 [85]	UK	RCT	NCDs risk	67† /142	38†	40-70 (IG: 54±0) (CG: 54±1)	10 weeks	Usual care	1.5	x	x	x
Taylor et al. 2020 [86]	UK	Pragmatic RCT	NCDs	232	152	18-75 (51±13)	12 months	Standard PARS	0	x	x	-
Van de Vijver et al. 2022 [87]	Netherlands	Uncontrolled follow-up study	NCDs or at risk	106	≥50	-	1 year	-	0	-	x	x
Ward et al. 2010 [88]	UK	Pre-post	NCDs	127† /279	187	24-88	12 months	-	0	x	-	x
Webb et al. 2016 [89]	UK	Cohort study	NCDs	11† /14	7	56±3	16 weeks	-	Mid-scheme	x	-	x
Williams et al. 2017 [90]	New Zealand	RCT	Type 2 diabetes	138	86	30-86	6 months	-	-	-	x	-

*PARS* physical activity referral scheme, *IG *intervention group, *CG *comparison group, *RCT *randomized controlled trial, *NCDs *noncommunicable diseases, *CVD *cardiovascular disease, *CHD* coronary heart disease, *TIA *Transient Ischemic Attack, the decimal numbers are reduced to integer

* The follow-up timepoint included in the analysis: harmonized starting at post-scheme = 0

† only the number of participants included in the analysis

‡ prescription/referral only

One-time: A single-session PARS. For example, during one appointment with the healthcare professional, the participant receives advice and a written prescription to follow independently

### Risk of bias in included studies

**Fig. 2:**
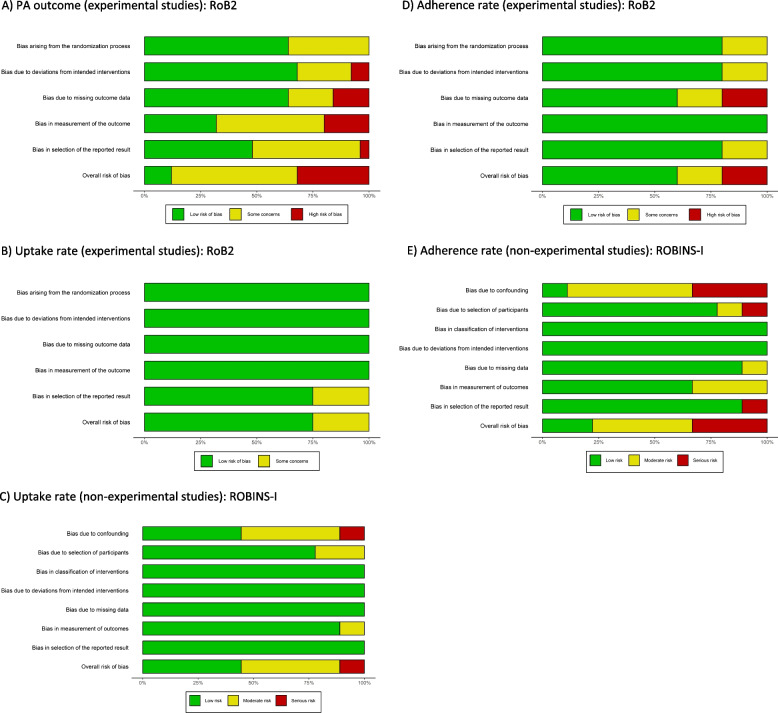
Risk of bias summary graphs across all the included studies classified according to the assessed outcome. †Cluster RCTs (k= 5) were rated additionally for bias arising from recruitment bias with ‘some concerns’, **A**) k = 25, **B**) k = 8, **C**) k = 9, **D**)k = 5,** E**) k = 9

Risk of bias assessment results for each outcome are summarized in Fig. 2. Detailed study-specific traffic light ratings are shown in Additional file 6. Most potential sources of bias in the RCTs were missing data, the measurement of PA outcome through self-report, and lack of pre-specified analysis protocols. The non-experimental studies pooled for uptake and adherence introduced a higher risk of bias.

### PARS effectiveness

#### PARS uptake

**Fig. 3:**
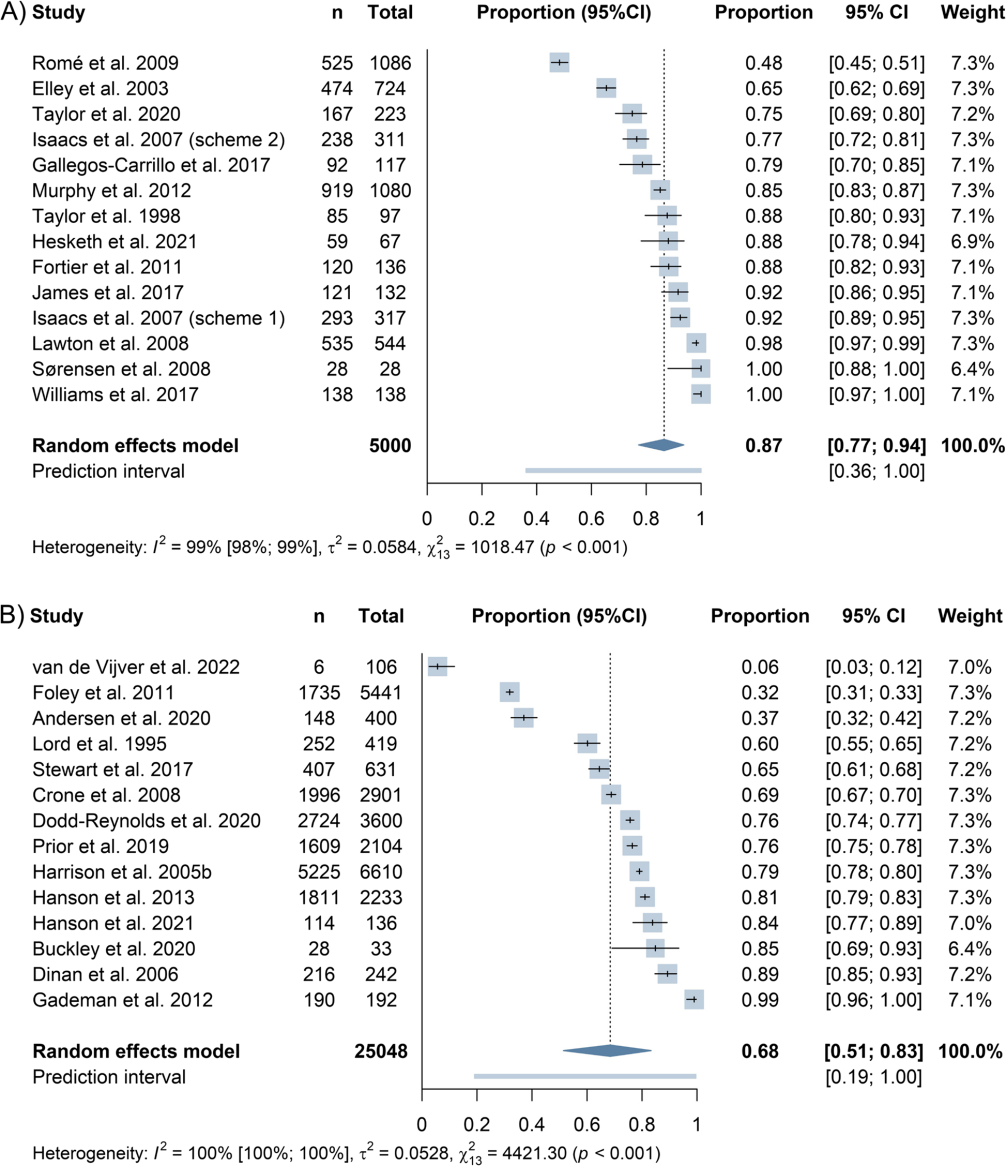
Forest plot of PARS uptake rate across experimental (**A**) and non-experimental studies (**B**) determined by proportional random-effects meta-analysis

On average, 87% (95%CI 77% to 94%) of individuals across 14 randomized trials and 68% (95%CI 51% to 83%) across 14 non-experimental studies that reported uptake opted to enter the offered PARS (Fig. 3). The heterogeneity statistics suggest that there is between-study variability in the true uptake rates. We found sub-group effect only for the analysis of non-experimental studies, where prescription schemes had lower uptake rate than referral schemes. However, unexplained heterogeneity remained extremely high (Additional file 7).

#### PARS adherence

**Fig. 4:**
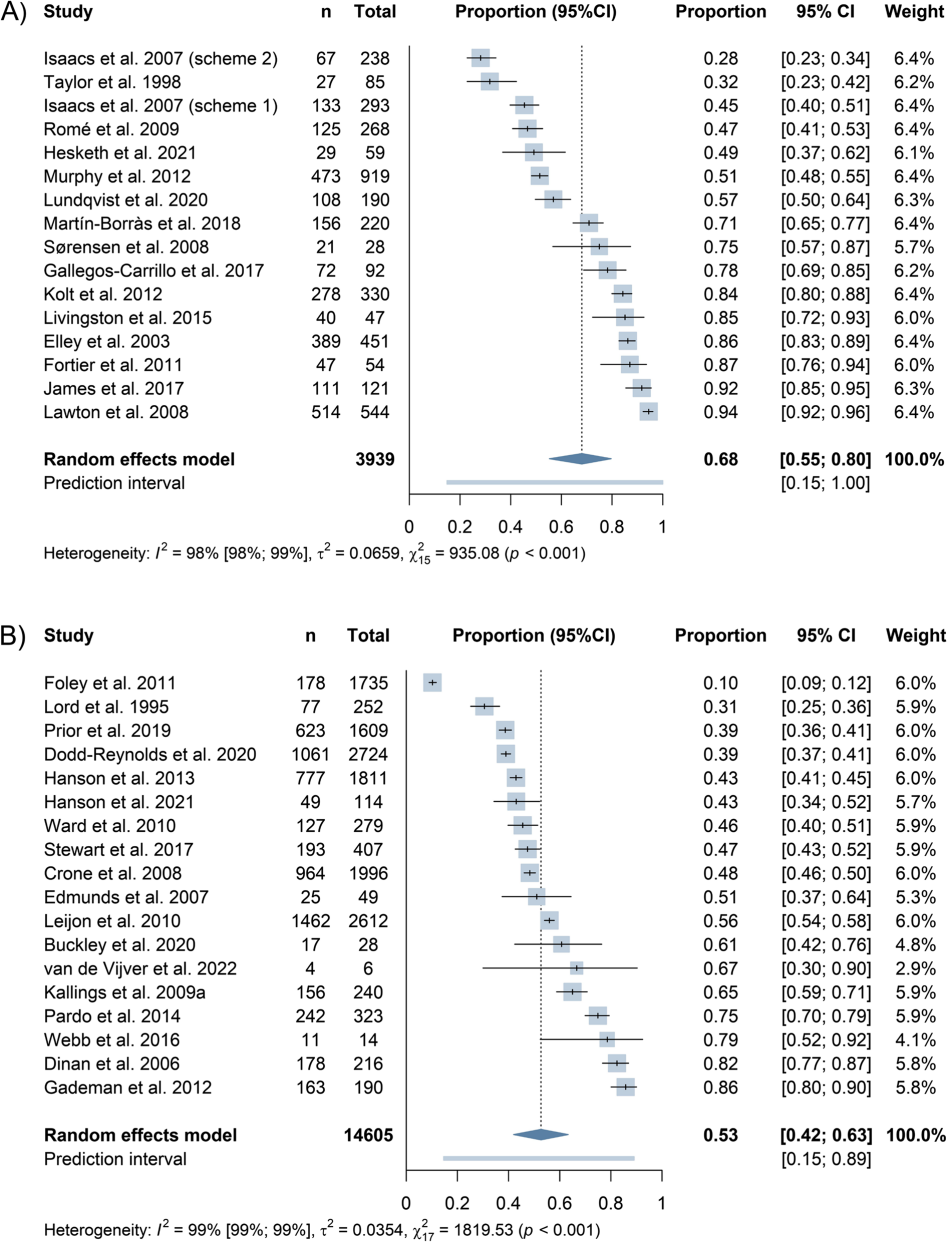
Forest plot of adherence to PARS across experimental (**A**) and non-experimental studies (**B**) determined by proportional random-effects meta-analysis

From those who took up PARS, 68% adhered to it (95%CI 55% to 80%) in experimental studies (Fig. 4). The pooled adherence rate among non-experimental studies was 53% (95%CI 42% to 63%). We found subgroup effects for risk of bias, location, and population only across experimental studies. High risk of bias, UK-based studies, referral schemes, or those including only at-risk populations reported the lowest adherence rates (Additional file 7). However, very high heterogeneity was still present.

#### Physical activity

**Fig. 5:**
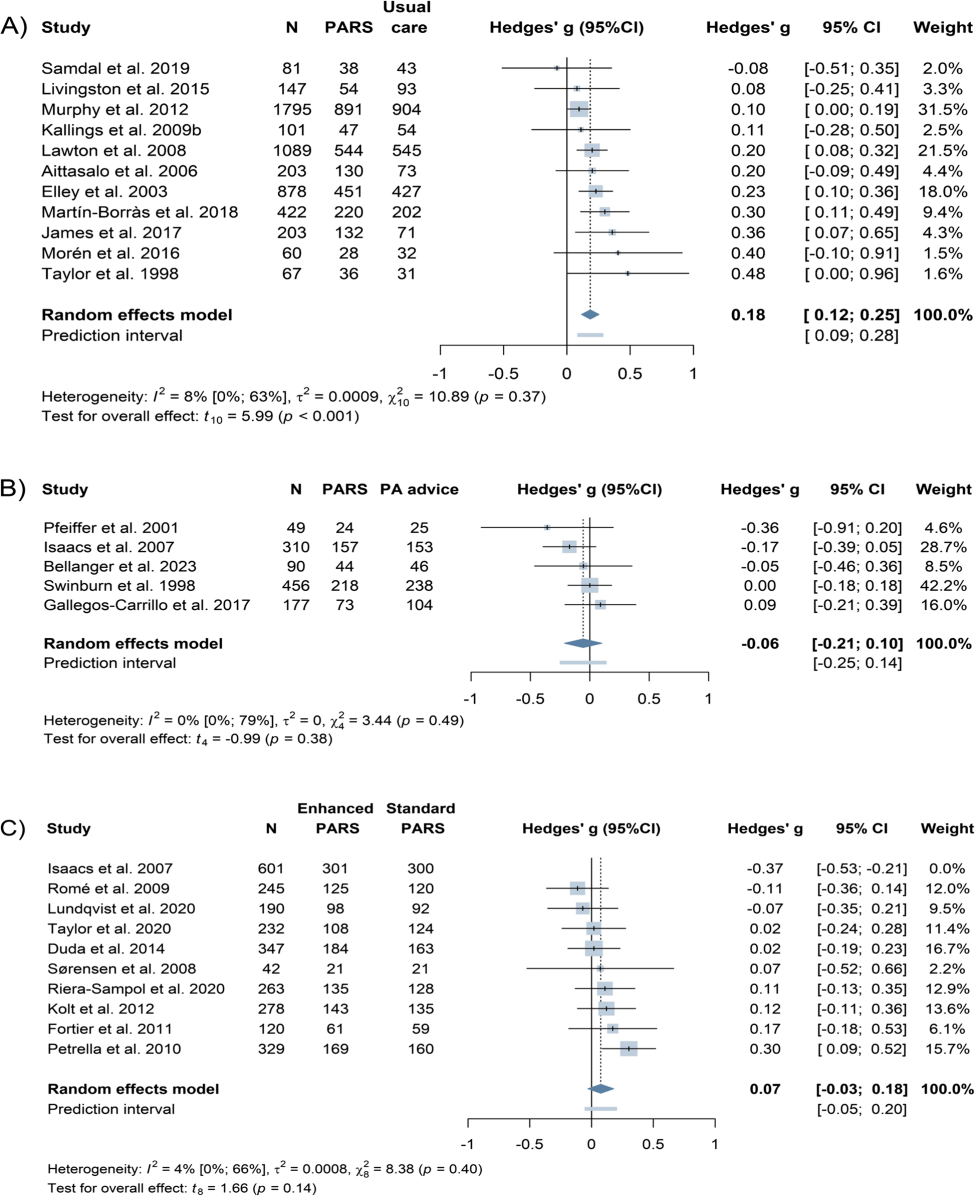
Forest plots indicating PARS effect on physical activity as compared to usual care, PA advice, and scheme intensity determined by random effects meta-analysis. Hedges’ g > 0 favors PARS, *PARS* physical activity referral scheme, *CI* confidence intervals, Meta-analysis **A** Omitting Murphy et al. 2012 as influential case for the PA analysis: g = 0.22, 95%CI 0.16 to 0.29, *p*-value < 0.0001, I^2^ = 0% [0.0%; 62.4%], Meta-analysis **B** Bellanger et al. 2023 included also active participants at baseline

The meta-analysis of 11 RCTs (*n* = 5046) showed that PA improved significantly in participants receiving PARS compared with usual care (Hedges’ g = 0.18, 95%CI 0.12 to 0.25), (Fig. 5). The magnitude of the effect was similar for objective (k = 5, g = 0.21, 95%CI -0.01 to 0.43) and subjective measures of PA (k = 6, g = 0.19, 95%CI 0.09 to 0.28). The NNT-Analysis showed that approximately 10 participants needed to receive PARS instead of usual care for one to increase their PA level. The pooled studies had low heterogeneity but with wide 95%CI (I^2^ = 8.2%, 95%CI 0.0% to 63.5%) and τ^2^ = 0.00 (95%CI 0.00 to 0.04). The prediction interval ranged from 0.09 to 0.28. The symmetrical funnel plot and Eggers’ test indicated no evidence of small-study effects (Additional file 8). The pooled effect size of RCTs comparing PARS with PA advice was g = -0.06 (95%CI -0.21 to 0.10). The observed heterogeneity was low (I^2^ = 0%, 95%CI 0.0% to 79%, τ^2^ = 0.00, 95%CI 0.00 to 0.17). Enhanced versions of PARS were not more effective than standard less intense models (g = 0.07, 95%CI -0.03 to 0.18). Twenty-four participants needed to follow an enhanced PARS, for one additional participant to increase their PA level compared to those who participated in a less intense version. This difference is not substantial and might be due to chance. The I^2^ statistic suggests that 4.5% of the observed between-study variability is due to true heterogeneity across the nine included studies (95%CI 0.0% to 66.4%). The study from Isaacs et al. [60] was identified as an outlier. Its inclusion in the analysis lowered the between-group difference to zero (g = 0.01, 95%CI -0.13 to 0.15) and increased heterogeneity to 70.8% (95%CI 44.3% to 84.7%). No publication bias was detected. Due to low heterogeneity no subgroup analysis was undertaken. Results for meta-analysis of specific PA types can be found in Additional file 9 and 10. Pooled non-experimental studies, with substantial heterogeneity and publication bias, showed a small to moderate effect of PARS on PA level (g = 0.40, 95%CI 0.14 to 0.66), (Additional file 11).

### Examination of PARS effectiveness by components

Because of an insufficient number of studies containing some of the components and/or their relevance to the outcome examined, we included 15 components in the meta-regression for PA, eight for uptake and 14 for adherence. No individual components predicted PA level or uptake in experimental studies (Table 3) or non-experimental studies (Additional file 12). Across all studies, PARS based on a person-centered approach or including screening or brief advice, reported 17% to 25% higher adherence rates. In contrast, offering PA sessions was negatively associated with adherence. However, the amount of unexplained heterogeneity remained substantially high.

**Table 3:** The relationship between PARS components and effect on physical activity level, uptake, and adherence rate determined by meta-regression

Component	PARS with the component	PARS without the component	Regression coefficient (B)	SE	*P* value	*R*^2^ (%)	I^2^ (%)
**PA level** (k = 25 RCTs)							
Person-centered approach	9	16	0.11	0.06	0.08	21.1	27.8
Individualized content	14	11	0.04	0.06	0.51	0.0	38.2
Behavior change theory	12	13	-0.03	0.06	0.60	0.0	35.5
Behavior change techniques	16	9	0.07	0.07	0.33	4.6	32.3
Screening	6	19	0.11	0.06	0.07	20.6	27.7
Brief advice	6	19	0.03	0.07	0.65	0.0	34.0
Written materials	6	19	-0.11	0.07	0.13	0.0	33.1
Written prescription	10	15	0.07	0.06	0.28	6.7	31.0
Referral	8	17	-0.01	0.07	0.93	0.0	35.6
Baseline consultation	13	12	0.00	0.06	0.95	0.0	35.4
Exit consultation	7	18	-0.10	0.06	0.13	17.6	27.7
Counseling support session(s)	8	17	0.01	0.07	0.88	0.0	36.6
Structured follow-up	7	18	0.07	0.06	0.26	0.0	32.9
PA sessions	9	16	-0.07	0.06	0.32	1.5	31.8
Exit strategies/routes	8	17	-0.06	0.07	0.35	0.0	32.9
*Number of components*	*-*	*-*	*-0.00*	*0.01*	*0.49*	*0.0*	*35.2*
**PARS uptake** (k = 28)							
Person-centered approach	10	18	0.12	0.11	0.29	0.0	99.5
Individualized content	21	7	0.08	0.12	0.53	0.0	99.6
Behavior change theory	9	19	0.07	0.11	0.56	0.0	99.5
Behavior change techniques	16	12	0.17	0.10	0.11	0.0	99.5
Screening	11	17	0.16	0.10	0.14	0.0	99.5
Brief advice	5	23	-0.02	0.14	0.90	0.0	99.5
Written prescription	13	15	0.06	0.52	0.61	31.3	99.2
Referral	20	8	-0.00	0.12	0.99	32.3	99.2
*Number of components*	*-*	*-*	*0.04*	*0.02*	0.02*	*3.72*	*99.5*
**PARS adherence** (k = 34)							
Person-centered approach	12	20	0.17	0.08	0.04*	21.5	98.7
Individualized content	26	8	0.05	0.10	0.60	0.0	99.1
Behavior change theory	14	20	0.10	0.08	0.25	0.0	99.1
Behavior change techniques	21	13	0.15	0.08	0.07	0.0	99.1
Screening	15	19	0.23	0.07	0.003**	30.4	98.6
Brief advice	8	26	0.25	0.09	0.008**	22.3	98.7
Written prescription	16	18	0.13	0.08	0.11	0.0	99.1
Referral	23	11	-0.06	0.09	0.48	0.0	99.1
Baseline consultation	19	15	-0.06	0.08	0.47	0.0	99.1
Exit consultation	15	19	-0.12	0.08	0.13	0.0	99.1
Counseling support session(s)	15	19	0.03	0.08	0.71	0.0	99.0
Structured follow-up	8	26	0.17	0.09	0.07	20.6	98.8
PA sessions	23	11	-0.20	0.08	0.02*	22.8	98.7
Action for non-attendance	5	29	-0.02	0.12	0.84	0.0	99.1
*Number of components*	*-*	*-*	*0.02*	*0.01*	*0.15*	*0.0*	*99.1*

Studies included in the PA meta-regression are RCTs. Studies included in uptake and adherence rate meta-regression are experimental and non-experimental combined, where also RCTs contribute with observational data

*Regression coefficient B* The difference in Hedges’ g between PARS with and without the component used as a predictor variable, *SE* Standard error of B, *p value* Significance of B, *R*^*2*^ The amount of heterogeneity explained from the component used as a predictor variable, *I*^*2*^ Residual heterogeneity not accounted for by the component, *n* Number of participants

^*^p < 0.05, **p < 0.01

The number of components in a PARS was identified as a predictor for uptake but not adherence and PA outcome. For any additional increase in number of components, the uptake rate is estimated to increase by around 6% (Table 3). However, the number of components accounts for a very small amount of heterogeneity.

### Certainty of evidence

We rated the certainty of evidence under the GRADE criteria for the PA meta-analysis comparing PARS to usual care as high. In contrast, our confidence in the pooled effect estimates for the comparison of PARS with PA advice or alternative PARS versions was limited. The proportional meta-analysis for uptake and adherence rates were descriptive in nature and characterized by very low certainty. See Additional file 13 for GRADE Evidence Profiles and Summary of Findings tables with detailed explanations of the rating decisions.

## Discussion

Our results show that PARS are more effective than usual care in increasing PA. We did not find any difference between PARS and PA advice only or various scheme intensities with regard to PA change. This was the first study to examine the potential role of PARS components in effectiveness. PARS components were regressed for their independent effect on PA, scheme uptake, and scheme adherence. Adherence was higher in PARS including a person-centered approach, screening, or brief advice, and lower in schemes offering PA sessions. The meta-regression did not detect a possible relationship between PARS components and PA level or uptake.

### Interpretation of the meta-analysis findings

Our study updates previous seminal meta-analyses and reinforces their the PA promoting effect of PARS [2, 16]. In our review, pooled data from 11 RCTs (*n* = 5046) showed that PARS result in a small increase in PA compared to usual care (high certainty of evidence). Approximately 10 PARS participants are needed for one to become more active. An earlier meta-analysis pooling five RCTs, concluded that 17 people need to participate for one to engage in moderate exercise [3]. However, the analysis pooled together all types of comparison groups [3]. Previous meta-analyses of PARS versus usual care reported that PARS participants had a 12% (95%CI 1.04 to 1.20, k = 5, *n* = 4504) [16] to 16% (95%CI 1.03 to 1.30, k = 4, *n* = 2334) [2] higher likelihood of achieving 90 to 150 min of at least moderate PA per week. While these statistics cannot be directly compared, they all confirm that adding PARS to usual care is associated with increased PA. Our meta-analysis adds that 95% of future studies comparing PARS with usual care may expect to have an effect size between 0.09 and 0.28. As with previous meta-analyses [2, 4, 16], we did not find any difference in PA level between PARS with PA advice only (5 RCTs) or with enhanced scheme versions (9 RCTs), (low certainty of evidence).

The lack of difference between the PARS and PA advice may be attributed to two key factors. Firstly, the PA advice demands a lesser commitment from participants compared to PARS. This may lead to lower uptake and adherence rates in PARS, ultimately fewer participants receiving the intervention as intended. Notably, one RCT revealed a significant difference in PA levels between the intervention and comparison groups when adherence rates exceeded 50% [54]. Secondly, participants in the PA advice group might increase their PA levels due to their participation in the study, a phenomenon known as the Hawthorne effect [39, 60]. We encountered similar arguments in the discussion sections of studies comparing standard PARS with enhanced versions, where no difference was detected. Enhanced scheme versions typically incorporate additional components or a higher session frequency, posing greater challenges to implementation by necessitating additional resources. For instance, a study that augmented the standard PARS using the Self-determination Theory reported additional difficulties in training scheme deliverers, potentially influencing the implementation of the enhanced intervention version [47].

As with Pavey et al. [17], experimental studies in our analysis reported significantly higher uptake levels than non-experimental studies and similar adherence in observational studies. However, Pavey et al. [17] reported much lower adherence across RCTs (49%, 95%CI 40% to 59%). Consistent with previous evidence [16, 17], we found considerable heterogeneity in uptake and adherence rates which could not be explained by subgroup analysis. However, the proportional data are known to be inherently highly heterogeneous and so this does not automatically signify data inconsistency [37].

### Interpretation of the meta-regression findings

This study is unique in that, to our knowledge, it is the first to examine associations between PARS components and PA, uptake, and adherence. However, the statistical power of the meta-regression was limited. We did not find significant associations between specific PARS components and PA level. Despite this, the regression coefficients indicate a greater effect on PA (g = 0.07 to 0.11) for schemes including a person-centered approach, behavior change techniques, screening, or a written prescription; and lower effect for schemes with exit consultations (g = -0.10). Although these effects appear to be negligible, they might have practical relevance in the context of overall small effect sizes observed in our PARS meta-analysis. Inability to reach statistical significance might be explained by not fulfilling the basic assumption of sufficient heterogeneity to carry out a meta-regression, which in our case was only 33.9% (95%CI 0.0% to 59.4%). Furthermore, we examined the impact of individual components rather than potential combinations. The added value of individual components on PA level was also investigated among some of the included studies. Findings regarding the counseling support session(s) [41, 51, 69] and written prescription [75, 84] were mixed. The inclusion of PA sessions [81] or basing the PARS on the Self-determination Theory [47] did not result in an added impact on PA level.

No component significantly predicted variation in PARS uptake. Prescription schemes reported approximately 6% higher uptake rates, but this relationship did not reach statistical significance and the amount of unaccounted heterogeneity remained substantially high. PARS including a person-centered approach, screening, or brief advice achieved higher adherence rates. In contrast, including PA sessions was associated with decreased adherence. While this is counterintuitive, Pavey et al. [17] also suggested that a higher number of sessions might be related to lower adherence. There are several potential explanations for this. PA session attendance provides a tangible measure of adherence that does not exist in PARS offering counseling only. Additionally, participants asked to attend PA sessions might face barriers related to transportation, accessibility, inconvenient timings, poor supervisory experiences, inadequate/inappropriate content, and lack of enjoyment, individualization, or relatable peers [3, 8, 56, 91]. PARS to date are based mainly on Social Cognitive Theory, Self-determination Theory, and the Transtheoretical Model [10], which give limited attention to the affective determinants of PA behavior such as enjoyment [92]. Future PARS could consider using the lens of affective science [93] to provide PA sessions that increase positive experiences and consequently engagement. PARS could intensify efforts towards increasing individual competencies needed for independent PA (e.g., PA-related Health Competence [94].

### Limitations and strengths

There are several caveats to this review. First, the results could be affected by the coding of components from scheme content reported in individual studies in our previous review [10]. To avoid subjective assumptions, we suggest authors of future studies identify and report PARS components based on our classification [10] and use the PARS taxonomy [95] to report characteristics. Second, not all components were investigated due to the limited number of studies available. Third, the relationship between components and PARS outcomes investigated through the meta-regression is not causal but observational [96]. The results might be misleading because of biases and confounding by other factors not related to scheme design (e.g., healthcare system characteristics) [97]. Fourth, we addressed only one aspect of PARS complexity: the components and a simplified linear relationship with scheme outcomes. Other characteristics of PARS and the causal pathway, such as between-components and scheme by context interactions, healthcare and societal ecosystems in which PARS are delivered, and characteristics of PARS delivers and receivers were not considered [18]. However, a focused question and simple analysis is suggested to be a good start for understanding complexity [18]. Fifth, the component content might be as important as whether the scheme includes it or not. For example, behavior change techniques may be implemented in varying degrees and combinations. Finally, only English and German publications were included, and the certainty of the evidence was assessed by one reviewer. This may introduce some uncertainties regarding the inclusion of all relevant studies and the confidence level of the pooled effect estimates.

The review also has several strengths. The methods were pre-registered and published to reduce bias or change of research question based on identified evidence. To avoid data dredging [96], meta-regression variables were prespecified in advance and we adhered to the prespecified question in the protocol [20]. All extracted data and analyses are transparent and reproducible. We included observational studies to provide naturalistic comparisons and rated the certainty of evidence for each meta-analysis outcome.

### Implications for practice and policy and future research

This study reinforces the potential of PARS as a strategy to support an active society by promoting PA in healthcare settings [1]. We highlight well-defined components that can guide PARS design. Consideration might be given to adding a person-centered approach, screening, or brief advice to existing schemes for improving the adherence rate. Future research should focus on understanding the role of components in PARS effectiveness. High quality experimental studies manipulating the use of components, such as factorial RCTs, could provide evidence about the effect of individual or combined components [98]. For example, in a two-by-two factorial experiment, two components, e.g., PA sessions and counseling support sessions, can be used as factors with two levels (present or absent). This results in four possible combinations to which participants can be randomly assigned. Additionally, research should compare the effect of components and their implementation cost. This could help optimize PARS by highlighting components that have a small effect but high implementation cost to help decision-makers find a balance between cost and effect. This is important to create sustainable PARS and increase their public health impact. Qualitative research exploring experiences of PARS participants and deliverers with the components could be valuable in contributing to wider understanding. Thus, mixed-methods designs are essential in evaluating PARS. The example of PARS and their complexity highlights that research about PA promotion in healthcare settings might benefit from the theories and methods used in complexity research [99] and systems thinking [100].

Our findings are hypothesis generating and not final conclusions. We encourage future studies to test the effect of the identified components. Further research is needed to confirm or establish new associations between PARS components and outcomes by using more sophisticated statistical methods such as component network meta-analysis models, and component individual participant data meta-analysis.

## Conclusions

Implementing PARS within healthcare settings might be valuable for effectively increasing PA on a broader scale. Findings from the meta-regression increase our understanding of the role of scheme components on PA, uptake and adherence. PARS may have higher adherence rates if they include a person-centered approach, screening, or brief advice. PARS including PA sessions reported lower adherence rates but as these are a promising source of PA experience, schemes should optimize the content of PA sessions and consider paying special attention to the affective response and enjoyment. No association was found between components and PA level or scheme uptake. However, components should not be disregarded because of statistical significance, but rather further investigated. Taken together, the findings indicate that scheme components can contribute to a better understanding of PARS effectiveness.

### Supplementary Information

Additional file 1. The updated search strategy.

Additional file 2. Microsoft Excel file with the formulas used to transform and harmonize the summary statistics reported across the included studies.

Additional file 3. Studies included in the previous systematic review that were excluded from the current analysis.

Additional file 4. Measurement of physical activity in the included studies.

Additional file 5. Definitions of uptake and adherence across the included studies.

Additional file 6. Traffic light plot for risk of bias assessment for physical activity, uptake, and adherence rate.

Additional file 7. Subgroup analysis results for PARS uptake and adherence.

Additional file 8. Funnel plots for the meta-analysis of physical activity outcome.

Additional file 9. Forest and funnel plots of PARS effect on various physical activity outcomes as compared to usual care or physical activity advice (RCTs).

Additional file 10. Meta-analysis of randomized trials comparing enhanced with standard PARS for specific physical activity outcomes.

Additional file 11. Meta-analysis of 12 non-experimental studies for physical activity level.

Additional file 12. The relationship between PARS components and effect on physical activity level from 12 pre-post studies.

Additional file 13. Summary of findings table and GRADE evidence profiles.

## Data Availability

The dataset and the R script used to generate the results in this article is available in the Open Science Framework repository at https://osf.io/dv8fb/?view_only=1703f57bd7f74c6ca0786e7093b531ec.

## Abbreviations

CI: Confidence interval
ES: Effect size
GRADE: Grading of Recommendations Assessment, Development and Evaluation
NNT: Number needed to treat
PA: Physical activity
PARS: Physical activity referral scheme(s)
RCT: Randomized controlled trial
RoB2: Cochrane risk-of-bias tool for randomized trials
ROBINS-I: Risk of Bias in Non-randomized Studies-of Interventions
SD: Standard deviation
SE: Standard error
