# Supplementary material for: Are physical activity referral scheme components associated with increased physical activity, scheme uptake, and adherence rate? A meta-analysis and meta-regression
Source: Int J Behav Nutr Phys Act. 2024 Aug 2;21:82. doi: 10.1186/s12966-024-01623-5 (PMC11295389; doi:10.1186/s12966-024-01623-5)
Supplement: Supplementary file 1 — Additional file 1. The updated search strategy. [file 12966_2024_1623_MOESM1_ESM.docx]

**Additional file 1.** The updated search strategy

| Date of search: | 03.11.2023 |  |
| --- | --- | --- |
| Filters: | Year 2023, English, German |  |
| Note: | The original search contained articles published from January 1990 to 2023 (Additional file 1, doi: 10.1186/s12966-023-01518-x). The search was updated on 03.11.2023. |  |
|  |  |  |
| *Source* | *Search Query* | *Hits* |
| **PubMed** | ((((((((exercise referral scheme*[Title/Abstract]) OR (exercise referral[Title/Abstract])) OR (exercise prescription schemes)) OR (exercise on prescription[Title/Abstract])) OR (physical activity prescription[Title/Abstract])) OR (physical activity referral scheme*[Title/Abstract])) OR (green prescription[Title/Abstract])) OR (physical activity prescription scheme)) OR (physical activity referral[Title/Abstract]) | 27 |
|  | ((((((motor activity[MeSH Terms]) OR (physical activit*[Title/Abstract])) OR (Exercise[MeSH Terms])) OR (exercise therapy[MeSH Terms])) OR (exercise[Title/Abstract])) AND ((((((counseling[MeSH Terms]) OR (referral and consultation[MeSH Terms])) OR (directive counseling[MeSH Terms])) OR (Prescriptions[MeSH Terms])) OR (prescription[Title/Abstract])) OR ((referral[Title/Abstract]) OR (prescribed physical activity[Title/Abstract])))) AND (((((primary health care[MeSH Terms]) OR (health promotion[MeSH Terms])) OR (physicians, primary care[MeSH Terms])) OR (general practi*[Title/Abstract])) OR (primary care intervention*[Title/Abstract])) | 1078 |
| **Scopus** | TITLE-ABS-KEY ( "exercise referral scheme*" ) OR TITLE-ABS-KEY ( {exercise referral} ) OR TITLE-ABS-KEY ( {exercise referral schemes} ) OR TITLE-ABS-KEY ( {exercise on prescription} ) OR TITLE-ABS-KEY ( {physical activity prescription} ) OR TITLE-ABS-KEY ( "physical activity referral scheme*" ) OR TITLE-ABS-KEY ( {green prescription} ) OR ALL ( {physical activity prescription scheme} ) OR TITLE-ABS-KEY ( {physical activity referral} ) | 30 |
|  | ( ( INDEXTERMS ( "Motor Activity" ) ) OR ( INDEXTERMS ( "physical activit*" ) ) OR ( INDEXTERMS ( "Exercise" ) ) OR ( TITLE-ABS-KEY ( exercise ) ) OR ( INDEXTERMS ( "Exercise Therapy" ) ) ) AND ( ( INDEXTERMS ( "counseling" ) ) OR ( ( "referral" AND INDEXTERMS ( "consultation" ) ) ) OR ( INDEXTERMS ( "prescriptions" ) ) OR ( TITLE-ABS-KEY ( "prescription*" ) ) OR ( TITLE-ABS-KEY ( "prescribed physical activit*" ) ) ) AND ( ( INDEXTERMS ( {primary health care} ) ) OR ( INDEXTERMS ( {health promotion} ) ) OR ( TITLE-ABS-KEY ( "primary care physician*" ) ) OR ( TITLE-ABS-KEY ( "general practi*" ) ) OR ( TITLE-ABS-KEY ( "primary care intervention*" ) ) ) | 102 |
| **Web of Science Core Collection** | ((((((((TS=("exercise referral scheme*")) OR TS=("exercise referral")) OR ALL=("exercise prescription scheme*")) OR TS=("exercise on prescription")) OR TS=("physical activity prescription")) OR TS=("physical activity referral scheme*")) OR TS=("green prescription")) OR ALL=(physical activity prescription scheme)) OR TS=("physical activity referral") | 19 |
|  | TS=("motor activit*" OR "physical activit*" OR exercise OR "exercise therap*") AND TS=(counsel$ing OR (referral AND consultation) OR "directive counsel$ing" OR "Motivational Interviewing" OR prescription* OR physical activit* NEAR/5 prescri*) AND TS=("primary health care" OR "health promotion" OR primary care physician* OR general practi* OR primary care NEAR intervention*) | 76 |
| **NIHR-HTA** | (exercise referral scheme*) OR (exercise on prescription) OR ((green prescription) OR (physical activity referral)) | 0 |
|  | ((MeSH DESCRIPTOR Motor Activity) OR (physical activity) OR (MeSH DESCRIPTOR Exercise) OR (MeSH DESCRIPTOR Exercise Therapy) OR (exercise)) AND ((MeSH DESCRIPTOR Counseling) OR (MeSH DESCRIPTOR Referral and Consultation ) OR (MeSH DESCRIPTOR Directive Counseling) OR (MeSH DESCRIPTOR Prescriptions) OR ((prescription) OR (referral)) OR (prescribed physical activit*)) AND ((MeSH DESCRIPTOR Primary Health Care) OR (MeSH DESCRIPTOR Health Promotion) OR (MeSH DESCRIPTOR Physicians, Primary Care) OR (general practi*) OR (primary care intervention*)) | 0 |
| **CINAHL** | ((((((((exercise referral scheme*[Title/Abstract]) OR (exercise referral[Title/Abstract])) OR (exercise prescription schemes)) OR (exercise on prescription[Title/Abstract])) OR (physical activity prescription[Title/Abstract])) OR (physical activity referral scheme*[Title/Abstract])) OR (green prescription[Title/Abstract])) OR (physical activity prescription scheme)) OR (physical activity referral[Title/Abstract]) | 0 |
|  | (( (MM "Physical Activity") OR (MM "Exercise") ) OR ( TI ( physicial activity or exercise ) OR AB ( physicial activity or exercise ) ) OR "exercise therapy") AND (( MM "Referral and Consultation" ) OR "prescription" OR ( TI prescription OR AB prescription ) OR ( TI ( referral or referral process or referral pathway or care pathway ) OR AB ( referral or referral process or referral pathway or care pathway ) ) ) AND (MM "Primary Health Care" OR MM "Secondary Health Care" OR MM "Health Promotion" OR MM "Physicians" ) | 16 |
| **CORE** | title:("exercise referral scheme*") abstract:("exercise referral scheme*") | 3 |
|  | title:("exercise referral") abstract:("exercise referral") | 5 |
|  | "exercise prescription scheme*" | 2 |
|  | title:("exercise on prescription") abstract:("exercise on prescription") | 1 |
|  | title:("physical activity prescription") abstract:("physical activity prescription") | 5 |
|  | title:("physical activity referral scheme*") abstract:("physical activity referral scheme*") | 0 |
|  | title:("green prescription") abstract:("green prescription") | 0 |
|  | title:("physical activity referral") abstract:("physical activity referral") | 0 |
| **Google Scholar** | allintitle: exercise referral scheme | 9 |
|  | allintitle: exercise referral | 17 |
|  | "exercise prescription scheme*" | 7 |
|  | allintitle: exercise on prescription | 16 |
|  | allintitle: physical activity prescription | 44 |
|  | allintitle: physical activity referral scheme | 1 |
|  | allintitle: green prescription | 3 |
|  | allintitle: physical activity prescription scheme | 0 |
|  | allintitle: physical activity referral | 10 |
| **Journals** | BMC Public Health | 3 |
|  | Scandinavian Journal of Public Health | 0 |
|  | British Journal of Sports Medicine | 8 |
|  | Scandinavian Journal of Medicine & Science in Sports | 0 |
|  | Australian and New Zealand Journal of Public Health | 0 |
|  | National Institute for Health and Care Excellence (NICE) | 0 |
