# Supplementary material for: Are physical activity referral scheme components associated with increased physical activity, scheme uptake, and adherence rate? A meta-analysis and meta-regression
Source: Int J Behav Nutr Phys Act. 2024 Aug 2;21:82. doi: 10.1186/s12966-024-01623-5 (PMC11295389; doi:10.1186/s12966-024-01623-5)
Supplement: Supplementary file 3 — Additional file 3. Studies included in the previous systematic review that were excluded from the current analysis. [file 12966_2024_1623_MOESM3_ESM.docx]

| **Additional file 3.** Studies included in the previous systematic review that were excluded from the current analysis | |
| --- | --- |
| **Reference** | **Reason for exclusion** |
| Bendrik R, Kallings LV, Bröms K, Kunanusornchai W, Emtner M. Physical activity on prescription in patients with hip or knee osteoarthritis: a randomized controlled trial. Clin Rehabil. 2021;35:1465-1477. doi:10.1177/02692155211008807. | Unable to compute Hedges’ d. Data reported as median and 95%CI. |
| Shepich J, Slowiak JM, Keniston A. Do subsidization and monitoring enhance adherence to prescribed exercise? Am J Health Promot. 2007;22:2–5. doi:10.4278/0890-1171-22.1.2. | Unable to harmonize the adherence rate presented as mean and SD. |
| Smith BJ, Bauman AE, Bull FC, Booth ML, Harris MF. Promoting physical activity in general practice: a controlled trial of written advice and information materials. Br J Sports Med. 2000;34:262–7. doi:10.1136/bjsm.34.4.262. | Missing SD. There was no similar included study to borrow the SD. |
| Kallings LV, Leijon M, Hellenius ML, Stahle A. Physical activity on prescription in primary health care: a follow-up of physical activity level and quality of life. Scand J Med Sci Sports. 2008;18:154–61. doi:10.1111/j.1600-0838.2007.00678.x. | PA data presented as categories which could not be converted to other units and were not sufficient to calculate Hedges’ g. |
| Leijon ME, Bendtsen P, Nilsen P, Festin K, Ståhle A. Does a physical activity referral scheme improve the physical activity among routine primary health care patients? Scand J Med Sci Sports. 2009;19:627–36. doi:10.1111/j.1600-0838.2008.00820.x. | PA data presented as categories which could not be converted to other units and were not sufficient to calculate Hedges’ g. |
| Rödjer L, H. Jonsdottir I, Börjesson M. Physical activity on prescription (PAP): self-reported physical activity and quality of life in a Swedish primary care population, 2-year follow-up. Scand J Prim Health Care. 2016;34:443–52. doi:10.1080/02813432.2016.1253820. | PA data presented as categories which could not be converted to other units and were not sufficient to calculate Hedges’ g. |

CI: confidence interval, SD: standard deviation
