# Supplementary material for: Are physical activity referral scheme components associated with increased physical activity, scheme uptake, and adherence rate? A meta-analysis and meta-regression
Source: Int J Behav Nutr Phys Act. 2024 Aug 2;21:82. doi: 10.1186/s12966-024-01623-5 (PMC11295389; doi:10.1186/s12966-024-01623-5)
Supplement: Supplementary file 4 — Additional file 4. Measurement of physical activity in the included studies. [file 12966_2024_1623_MOESM4_ESM.docx]

| **Additional file 4.** Measurement of physical activity in the included studies | | | | |
| --- | --- | --- | --- | --- |
| **Study** | **PA measure** | **PA domains** | **Validity** | **PA recall** |
| Aittasalo et al.  2006 [39] | Self-report: IPAQ short version† | All daily PA excluding work | - | 7 days |
| Andersen et al.  2020 [40] | Self-report: Swedish National Board of Health and Welfare Questions | Overall, everyday activities, exercise | - | Weekly |
| Bellanger et al. 2023 [38] | Self-report: IPAQ | Total PA | Yes | Last 7 days |
|  | Devise measured: Actigraph wGT3x-BT | Total PA | Yes | 7 days |
| Bredahl et al.  2011 [41] | Self-report: questionnaire | Total PA | - | 6 months |
| Buckley et al.  2020 [42] | Devise measured: ActiGraph GT3X/GT1M | Light, moderate, and vigorous PA | Yes | 7 days |
| Dodd-Reynolds et al. 2020 [45] | Self-report: GLTEQ | Leisure exercise | Yes | Typical week |
| Duda et al.  2014 [46] | Self-report: 7-Day Physical Activity Recall† | Any PA of at least moderate intensity | Yes | Past week |
| Elley et al.  2003 [48] | Self-report: questionnaire from the Auckland heart study | Leisure exercise, leisure PA, total leisure time EE | Yes | Over 3 months |
| Fortier et al.  2011 [50] | Self-report: GLTEQ | Leisure time | Yes | Typical week |
|  | Devise measured: Actical, Mini Mitter Co., Inc., Bend, Ore | MVPA, moderate PA, vigorous PA | Yes | 14 days |
| Gademan et al.  2012 [51] | Self-report: SQUASH questionnaire† | Total PA, leisure time | Yes | Not reported |
| Galaviz et al.  2013 [52] | Self-report: GLTEQ modified | Leisure time PA | Yes | Typical week |
| Gallegos-Carrillo et al. 2017 [53] | Self-report: IPAQ | Total PA | Yes | Last 7 days |
|  | Devise measured: ActiGraph GT3X/GT1M | MVPA | Yes | 7 days |
| Hanson et al.  2013 [54] | Self-report: GLTEQ | Leisure exercise | Yes | Typical week |
| Hanson et al.  2021 [55] | Self-report: GPAQ | Total PA | Yes | Typical week |
| Harrison et al.  2005a [57] | Self-report: 7D PAR | MVPA | Yes | Past week |
| Isaacs et al.  2007 [59] | Self-report: 7D PAR | MVPA, total PA | No | 7-day |
| James et al.  2017 [60] | Devise measured: G-Sensor 2025 pedometer | Walking | Yes | 7 days |
| Kallings et al.  2009b [61] | Self-report: Seven consecutive day diary | MVPA | - | 7 days |
|  | Device-measured: Yamax Digiwalker SW-200 | Daily steps | - | 7 days |
| Kolt et al.  2012 [63] | Self-report: Auckland Heart Study Physical Activity Questionnaire | Total PA, MVPA, walking | Yes | Over 3 months |
| Lawton et al.  2008 [64] | Self-report: NZPAQ-SF | Total PA | Yes | Past 7 days |
| Livingston et al.  2015 [66] | Self-report: GLTEQ | MVPA, moderate PA, vigorous PA | Yes | Average week |
|  | Devise measured: ActiGraph GT3X/GT1M | MVPA, moderate PA, vigorous PA | Yes | 7 days |
| Lundqvist et al.  2020 [68] | Self-report: IPAQ | Total PA | Yes | Previous week |
| Martín-Borràs et al. 2018 [69] | Self-report: IPAQ short form | Total PA | Yes | Last 7days |
| Morén et al.  2016 [70] | Devise measured: ActiGraph GT3X/GT1M | MVPA, Walking | Yes | na |
| Murphy et al.  2012 [71] | Self-report: 7-D PAR† | Leisure and occupational | Yes | Past 7 days |
| Pardo et al.  2014 [72] | Self-report: IPAQ short form† | Total PA | Yes | Last 7 days |
| Petrella et al.  2010 [73] | Self-report: 7-Day Physical Activity Recall | Total PA | Yes | 7-day |
| Pfeiffer et al.  2001 [74] | Self-report: not specified (adapted for older adults from Swinburn et al. 1998) | Total PA, leisure, walking | No | Over the previous two weeks |
| Prior et al.  2019 [75] | Self-report: IPAQ short form | Total PA | Yes | Last 7 days |
| Riera-Sampol et al. 2020 [76] | Self-report: IPAQ short form | Total PA | Yes | Last 7 days |
| Romé et al.  2009 [77] | Self-report: IPAQ short version | Total PA | - | Last 7 days |
| Samdal et al.  2019 [78] | Devise measured: SenseWear Pro Armband Mini; BodyMedia Inc., Pittsburgh, Pennsylvania, USA | MVPA | Yes | 7 days |
| Sjöling et al.  2011 [79] | Self-report: Exercise diary | Leisure exercise | - | Daily exercise diary |
| Sørensen et al.  2008 [80] | Self-report: ‘‘How many days during an average week are you physically active 30 min or more?” | Total PA | No | Average week |
| Sørensen et al.  2011 [81] | Self-report: several short,  previously validated items | Total PA | Yes | - |
| Stewart et al.  2017 [82] | Self-report: GPPAQ | Total PA | Yes | Last week |
| Swinburn et al.  1998 [83] | Self-report: not specified | Leisure PA | Yes | Over the previous two weeks |
| Taylor et al.  1998 [84] | Self-report: Blair’s seven day recall method | Total PA, moderate PA, vigorous PA | - | 7-day |
| Taylor et al.  2020 [85] | Devise measured: GENEActiv™ Original accelerometer (version 3.009.02.2015) | MVPA | Yes | 10 days |
|  | Self-report: 7-Day Physical Activity Recall | MVPA | Yes | 7-day |
| Ward et al.  2010 [87] | Self-report: self-reported ‘sessions’ of 30 minutes of moderate or 20 minutes of vigorous activity accumulated as 10 minutes or more | MVPA | No | - |
| Webb et al.  2016 [88] | Self-report: IPAQ | Total PA | Yes | Last 7 days |
|  | Devise measured: SenseWear Pro Armband Mini; BodyMedia Inc., Pittsburgh, Pennsylvania, USA | Walking | Yes | na |

PA: physical activity, MVPA: moderate to vigorous physical activity, EE: energy expenditure, Total PA: leisure time, domestic and gardening (yard) activities, work-related and transport-related activity (unless otherwise indicated), IPAQ: International Physical Activity Questionnaire, SQUASH: Short Physical Activity Questionnaire with the General Purpose to Assess Habitual Physical Activity, GLTEQ: Godin Leisure Time Exercise Questionnaire, 7-D PAR: 7-Day Physical Activity Recall, NZPAQ-SF: New Zealand Physical Activity Questionnaire Short Form, SGPALS: Saltin-Grimby Physical Activity Level Scale, GPPAQ: General Practice Physical Activity Questionnaire

† Interviewer-administered questionnaire

- Not stated or unclear
