# Supplementary material for: Are physical activity referral scheme components associated with increased physical activity, scheme uptake, and adherence rate? A meta-analysis and meta-regression
Source: Int J Behav Nutr Phys Act. 2024 Aug 2;21:82. doi: 10.1186/s12966-024-01623-5 (PMC11295389; doi:10.1186/s12966-024-01623-5)
Supplement: Supplementary file 5 — Additional file 5. Definitions of uptake and adherence across the included studies. [file 12966_2024_1623_MOESM5_ESM.docx]

| **Additional file 5.** Definitions of uptake and adherence across the included studies | | |
| --- | --- | --- |
| **Study** | **Uptake definition** | **Adherence definition** |
| Andersen et al. 2020 [40] | Participants who used counsellor support. | na |
| Buckley et al. 2020 [42] | Participants attending the first consultation session (induction) after referral. | Participants attending the 12 week consultation. |
| Crone et al. 2008 [43] | Participants who attended their first session. | Those who took up the scheme and attended ≥80% of the supervised exercise sessions (completers). |
| Dinan et al. 2006 [44] | Those who took up the referral. | From those referred, the number of participants who completed the cycle of exercise classes.† |
| Dodd-Reynolds et al. 2020 [45] | Those who started the scheme after receiving the referral. | Those who took up and completed the scheme. |
| Edmunds et al. 2007 [47] | na | Participants who were still exercising at the end of the EoP in accordance with their prescriptions. |
| Elley et al. 2003 [48] | Inactive eligible persons who agreed to enter the scheme by participating in the study.† | Those completing 12 months follow up. |
| Foley et al. 2011 [49] | Those responding to initial contact by GRx patient support personnel and registered in the GRx program. | Participants completing the GRx program. |
| Fortier et al. 2011 [50] | Those eligible for randomization to intensive counseling or no intervention, and did show up at enrollment. | Those who attended all the six intensive counseling sessions. |
| Gademan et al. 2012 [51] | Those taking up the first supervised exercise session. | Participants who finished the program and attended almost all 18 exercise sessions. |
| Gallegos-Carrillo et al. 2017 [53] | Participants enrolled in the exercise referral program and showed up at the center or completed eligibility assessment. | Those attending ≥ 50% (24 of 48 total) of the planned sessions. |
| Hanson et al. 2013 [54] | Number of participants admitted to the scheme following the pre-scheme consultation | Number of participants who attended the 24-week consultation |
| Hanson et al. 2021 [55] | Those who attended at least one PARS session after referral. | Those who took up the referral and attended 8 or more sessions (≤ 67% of the total sessions) during the 12-week program. |
| Harrison et al. 2005b [56] | Those referred to the exercise officer and attended at least the first consultation. | na |
| Hesketh et al. 2021 [58] | Participants who completed at least one training session. | Participants who completed the scheme |
| Isaacs et al. 2007 [59] | Those who were referred to the leisure centre (referrals received at the centre) and attended at least one session in the leisure centre. | Those attending at least 75 % of the exercise sessions. |
| James et al. 2017 [60] | Participants who were referred and randomized to the intervention group and attended at least one session.† | Those attending at least 4 out of 5 sessions. |
| Kallings et al. 2009a [62] | na | Participants who adhered to the prescribed PA. |
| Kolt et al. 2012 [63] | na | Those who finished the intervention. |
| Lawton et al. 2008 [64] | Those who attended telephone counseling. | Participants who received the full intervention. |
| Leijon et al. 2010 [65] | na | Participants who adhered to the prescribed PA. |
| Livingston et al. 2015 [66] | na | Those who completed the supervised exercise program and adhered to at least 18 of the 24 gym-based sessions. |
| Lord et al. 1995 [67] | Those attending an initial consultation with the community health fitness officer. | Participants who returned to attend a ten-week consultation and who were still exercising (completed). |
| Lundqvist et al. 2020 [68] | na | Those who adhered to the allocated intervention. |
| Martín-Borràs et al. 2018 [69] | na | Those attending at 19 out of 24 sessions. |
| Murphy et al. 2012 [71] | Those who attended at least one NERS session after referral.† | Participants completing the 16 weeks PARS. |
| Pardo et al. 2014 [72] | na | Those who completed the scheme. |
| Prior et al. 2019 [75] | From those referred, the ones who entered the scheme. | Those who completed the scheme. |
| Romé et al. 2009  [77] | Participants who choose to continue the program after being offered.† | Those who completed of the 4-month program. |
| Sørensen et al. 2008 [80] | Number randomized to the intervention group and started intervention. | Participation rate in the counseling sessions. |
| Stewart et al. 2017  [82] | Referred participants attending baseline assessment. | Those who started (attended baseline assessment) and attended the post assessment. |
| Taylor et al. 1998  [84] | Persons referred who used the prescription. | Attended at least 15 out of the 20 prescribed sessions. |
| Taylor et al. 2020  [85] | Those who attended the scheme at least once. | na |
| van de Vijver et al. 2022  [86] | Those who participated at least once in the peer coach PA intervention where they were referred. | A referred person who participated once and did not dropout during the study period. |
| Ward et al. 2010  [87] | na | Those still in the program after 12 months. |
| Webb et al. 2016  [88] | na | Attendance of the prescribed exercise sessions. |
| Williams et al. 2017  [89] | Uptake into the study and into the service/program. | na |

na: not applicable

† indicates our interpretation
