# Supplementary material for: Are physical activity referral scheme components associated with increased physical activity, scheme uptake, and adherence rate? A meta-analysis and meta-regression
Source: Int J Behav Nutr Phys Act. 2024 Aug 2;21:82. doi: 10.1186/s12966-024-01623-5 (PMC11295389; doi:10.1186/s12966-024-01623-5)
Supplement: Supplementary file 6 — Additional file 6. Traffic light plot for risk of bias assessment for physical activity, uptake, and adherence rate. [file 12966_2024_1623_MOESM6_ESM.docx]

**Additional file 6.** Traffic light plot for risk of bias assessment for physical activity, uptake, and adherence rate

| A) Experimental studies assessed with RoB2 | B) Non-experimental studies assessed with ROBINS-I |
| --- | --- |
|  | 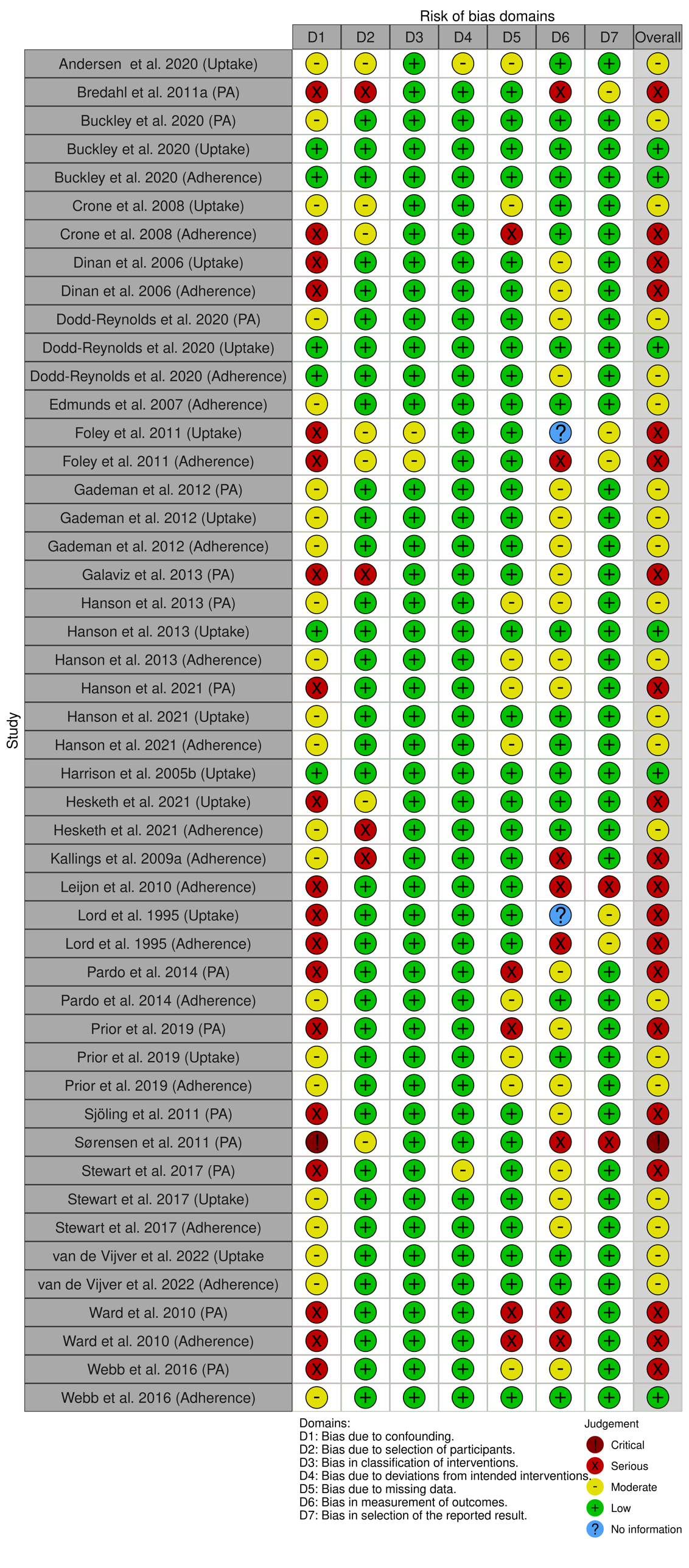 |
| † Cluster RCTs rated additionally for bias arising from the identification/recruitment of participants: all as ‘some concerns’ |  |
