# Supplementary material for: Are physical activity referral scheme components associated with increased physical activity, scheme uptake, and adherence rate? A meta-analysis and meta-regression
Source: Int J Behav Nutr Phys Act. 2024 Aug 2;21:82. doi: 10.1186/s12966-024-01623-5 (PMC11295389; doi:10.1186/s12966-024-01623-5)
Supplement: Supplementary file 7 — Additional file 7. Subgroup analysis results for PARS uptake and adherence. [file 12966_2024_1623_MOESM7_ESM.docx]

**Additional file 7.** Subgroup analysis results for PARS uptake and adherence

|  |  | k | Proportion | 95% CI | I^2^ (%) | P _subgroup_ |
| --- | --- | --- | --- | --- | --- | --- |
| Uptake rate | | | | | | |
| Risk of bias | |  |  |  |  | 0.68 |
| *Experimental studies* | |  |  |  |  |  |
|  | Low to moderate | 9 | 0.85 | 0.76-0.92 | 99.0 |  |
|  | High | 5 | 0.89 | 0.57-1.00 | 97.8 |  |
| *Non-experimental studies* | |  |  |  |  | 0.20 |
|  | Low to moderate | 6 | 0.79 | 0.53-0.96 | 98.8 |  |
|  | High | 8 | 0.60 | 0.34-0.84 | 99.7 |  |
| Location^1^ | |  |  |  |  |  |
| *Experimental studies^1^* | |  |  |  |  | 0.39 |
|  | UK | 8 | 0.84 | 0.69-0.94 | 98.6 |  |
|  | Outside Europe | 6 | 0.90 | 0.73-0.99 | 98.7 |  |
| Population | |  |  |  |  |  |
| *Experimental studies^1^* | |  |  |  |  | 0.83 |
|  | At risk of NCDs | 3 | 0.86 | 0.62-0.99 | 93.8 |  |
|  | With or at risk of NCDs | 4 | 0.91 | 0.57-1.00 | 99.1 |  |
|  | With NCDs | 7 | 0.84 | 0.66-0.97 | 98.7 |  |
| Scheme type | |  |  |  |  |  |
| *Experimental studies* | |  |  |  |  | 0.11 |
|  | Prescription scheme^2^ | 5 | 0.94 | 0.72-1.00 | 99.0 |  |
|  | Referral scheme^3^ | 9 | 0.81 | 0.71-0.90 | 98.7 |  |
| *Non-experimental studies* | |  |  |  |  | 0.006** |
|  | Prescription scheme^2^ | 3 | 0.43 | 0.11-0.79 | 98.5 |  |
|  | Referral scheme^3^ | 11 | 0.75 | 0.57-0.90 | 98.4 |  |
| PARS length | |  |  |  |  |  |
| *Experimental studies* | |  |  |  |  | 0.72 |
|  | Up to 13 weeks | 7 | 0.85 | 0.76-0.92 | 96.0 |  |
|  | > 13 weeks | 7 | 0.88 | 0.67-0.99 | 99.3 |  |
| *Non-experimental studies* | |  |  |  |  | 0.39 |
|  | Up to 13 weeks | 6 | 0.75 | 0.62-0.86 | 97.8 |  |
|  | > 13 weeks | 8 | 0.63 | 0.31-0.90 | 99.8 |  |
| Adherence rate | | | | | | |
| Risk of bias | |  |  |  |  |  |
| *Experimental studies* | |  |  |  |  | 0.03* |
|  | Low to moderate | 12 | 0.73 | 0.59-0.86 | 98.7 |  |
|  | High | 4 | 0.50 | 0.23-0.77 | 82.5 |  |
| *Non-experimental studies* | |  |  |  |  | 0.65 |
|  | Low to moderate | 5 | 0.56 | 0.31-0.80 | 97.9 |  |
|  | High | 13 | 0.51 | 0.38-0.65 | 99.3 |  |
| Location | |  |  |  |  |  |
| *Experimental studies* | |  |  |  |  | <0.001*** |
|  | Europe | 9 | 0.50 | 0.38-0.62 | 93.1 |  |
|  | *UK* | *5* | *0.41* | *0.28-0.55* | *92.2* |  |
|  | *Non-UK* | *4* | *0.61* | *0.40-0.80* | *91.0* |  |
|  | Outside Europe | 7 | 0.87 | 0.82-0.92 | 85.7 |  |
| *Non-experimental studies* | |  |  |  |  |  |
|  | Europe, UK | 12 | 0.49 | 0.39-0.58 | 95.4 |  |
|  | Europe, non-UK | 5 | 0.71 |  |  |  |
| Population | |  |  |  |  |  |
| *Experimental studies* | |  |  |  |  | <0.001*** |
|  | At risk of NCDs | 4 | 0.40 | 0.21-0.62 | 92.8 |  |
|  | With or at risk of NCDs | 5 | 0.80 | 0.57-0.96 | 99.1 |  |
|  | With NCDs | 7 | 0.74 | 0.56-0.89 | 95.3 |  |
| *Non-experimental studies* | |  |  |  |  | 0.60 |
|  | At risk of NCDs | 2 | 0.50 | 0.00-1.00 | 92.1 |  |
|  | With or at risk of NCDs | 10 | 0.57 | 0.38-0.75 | 99.5 |  |
|  | With NCDs | 6 | 0.48 | 0.41-0.54 | 65.2 |  |
| Scheme type | |  |  |  |  |  |
| *Experimental studies* | |  |  |  |  | 0.01* |
|  | Prescription scheme^2^ | 6 | 0.82 | 0.67-0.94 | 96.3 |  |
|  | Referral scheme^3^ | 10 | 0.59 | 0.41-0.75 | 96.7 |  |
| *Non-experimental studies* | |  |  |  |  | 0.18 |
|  | Prescription scheme^2^ | 5 | 0.41 | 0.14-0.71 | 99.7 |  |
|  | Referral scheme^3^ | 13 | 0.58 | 0.46-0.69 | 97.6 |  |
| PARS length | |  |  |  |  |  |
| *Experimental studies* | |  |  |  |  | 0.94 |
|  | Up to 13 weeks | 10 | 0.68 | 0.49-0.84 | 98.1 |  |
|  | > 13 weeks | 6 | 0.69 | 0.46-0.88 | 98.9 |  |
| *Non-experimental studies* | |  |  |  |  | 0.89 |
|  | Up to 13 weeks | 8 | 0.53 | 0.40-0.67 | 96.4 |  |
|  | > 13 weeks | 10 | 0.52 | 0.33-0.70 | 99.3 |  |

K: number of studies, CI: confidence intervals, I^2^: level of statistical heterogeneity within the subgroup

^1^ No sub-group analysis for non-experimental studies due to inappropriate number of studies per subgroup (k<2)

^2^ Includes written prescription and no referral form

^3^ Includes referral, and might include additional prescription

* p<0.05, ** p<0.01, *** p<0.001
