# Supplementary material for: Are physical activity referral scheme components associated with increased physical activity, scheme uptake, and adherence rate? A meta-analysis and meta-regression
Source: Int J Behav Nutr Phys Act. 2024 Aug 2;21:82. doi: 10.1186/s12966-024-01623-5 (PMC11295389; doi:10.1186/s12966-024-01623-5)
Supplement: Supplementary file 8 — Additional file 8. Funnel plots for the meta-analysis of physical activity outcome. [file 12966_2024_1623_MOESM8_ESM.docx]

**Additional file 8.** Funnel plots for the meta-analysis of physical activity outcome





Eggers’ test: k=11, intercept ^β0=0.661, 95%CI -0.5 to - 1.82, t=1.117, p=0.29


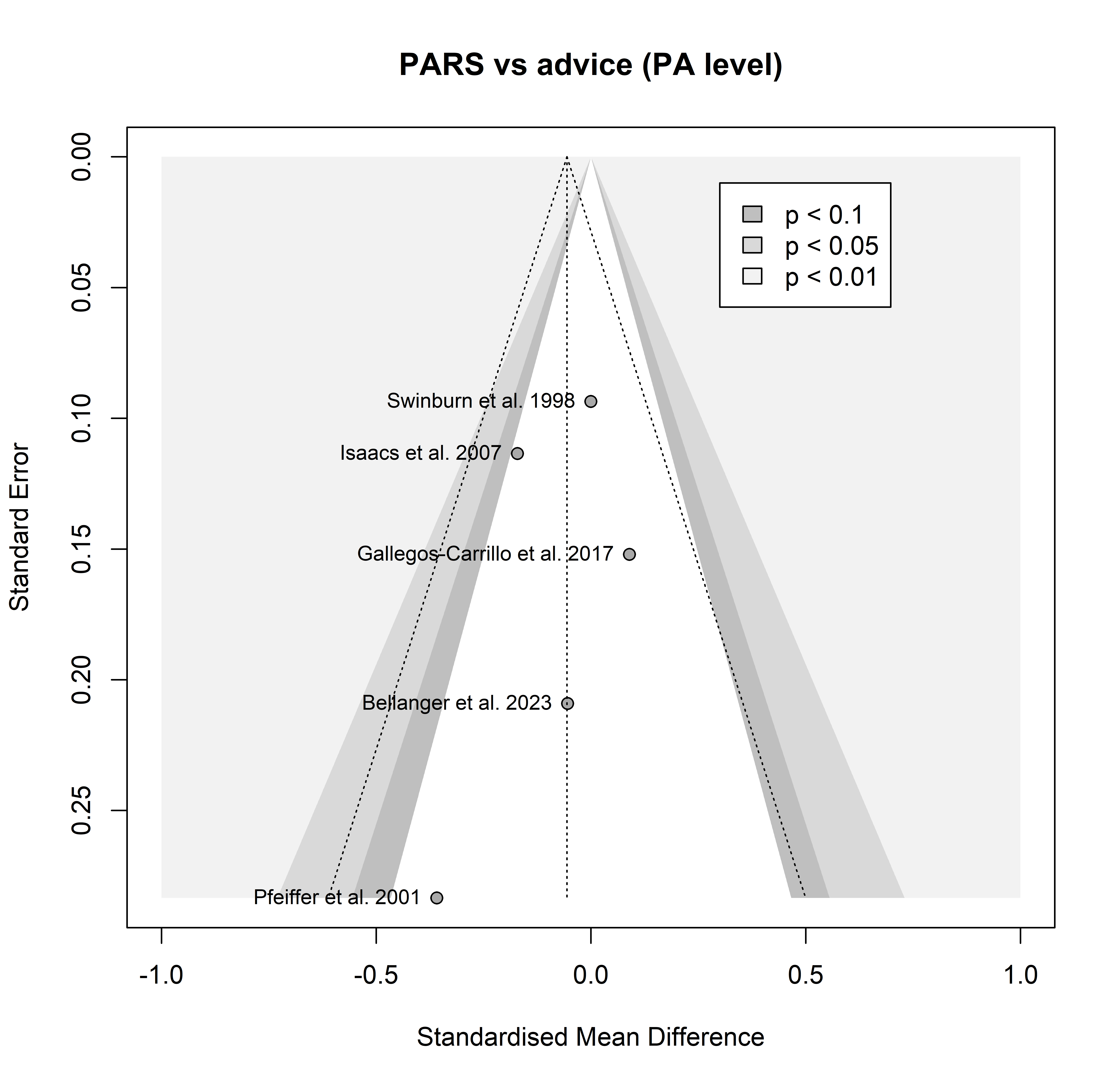


k=5





K=9
