# Supplementary material for: Are physical activity referral scheme components associated with increased physical activity, scheme uptake, and adherence rate? A meta-analysis and meta-regression
Source: Int J Behav Nutr Phys Act. 2024 Aug 2;21:82. doi: 10.1186/s12966-024-01623-5 (PMC11295389; doi:10.1186/s12966-024-01623-5)
Supplement: Supplementary file 9 — Additional file 9. Forest and funnel plots of PARS effect on various physical activity outcomes as compared to usual care or physical activity advice (RCTs). [file 12966_2024_1623_MOESM9_ESM.docx]

**Additional file 9.** Forest and funnel plots of PARS effect on various physical activity outcomes as compared to usual care or physical activity advice (RCTs)

**A) PARS vs usual care**

**Total PA**


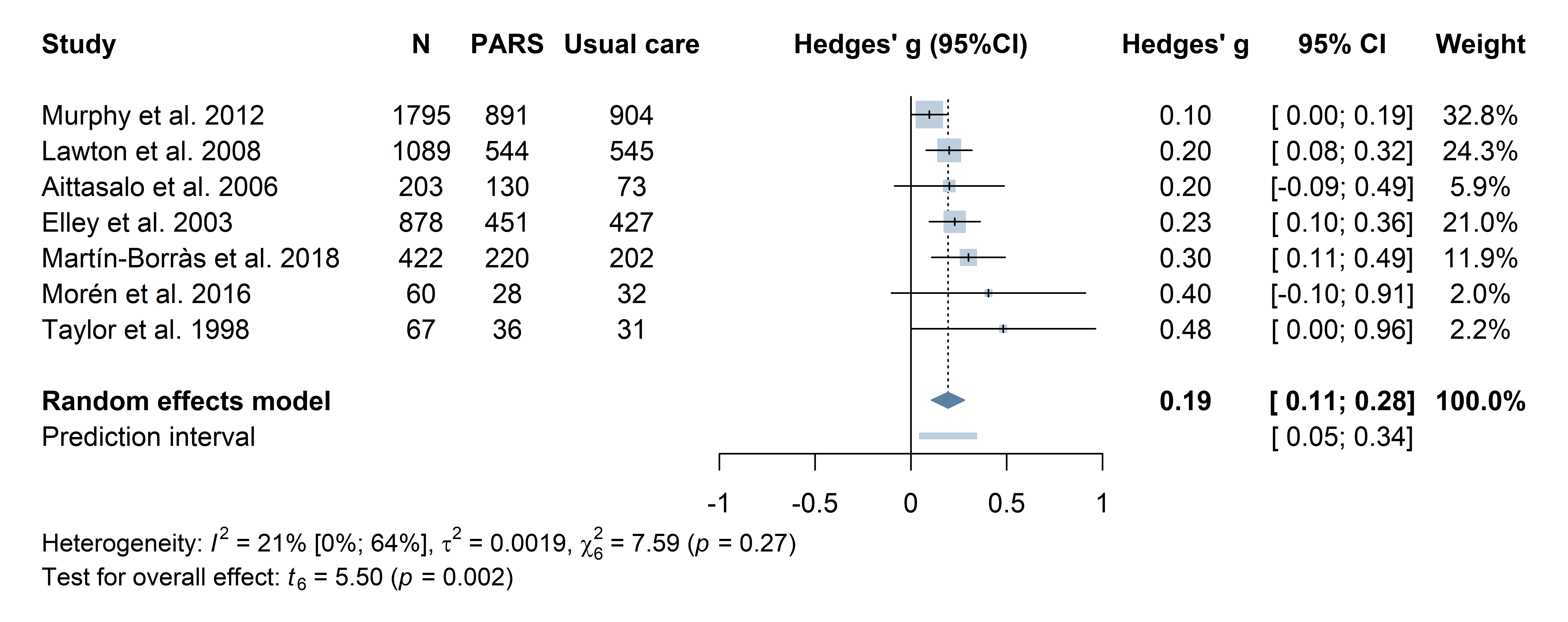


**Moderate to vigorous PA**


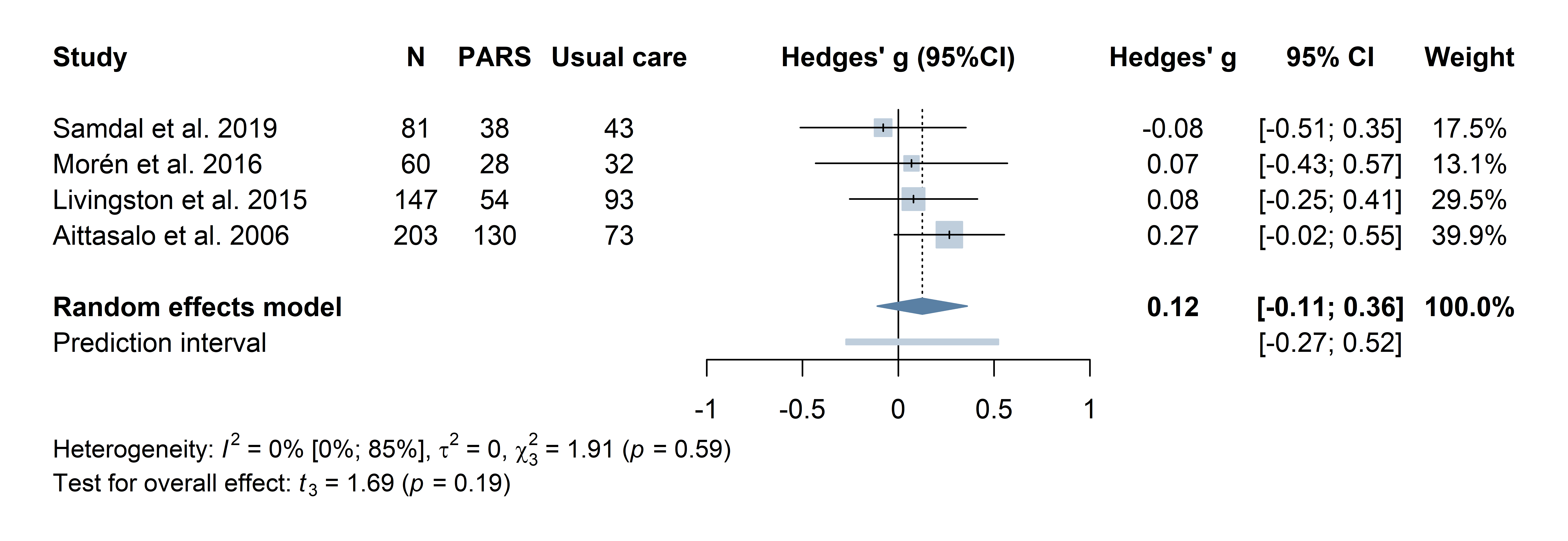


**Vigorous PA**


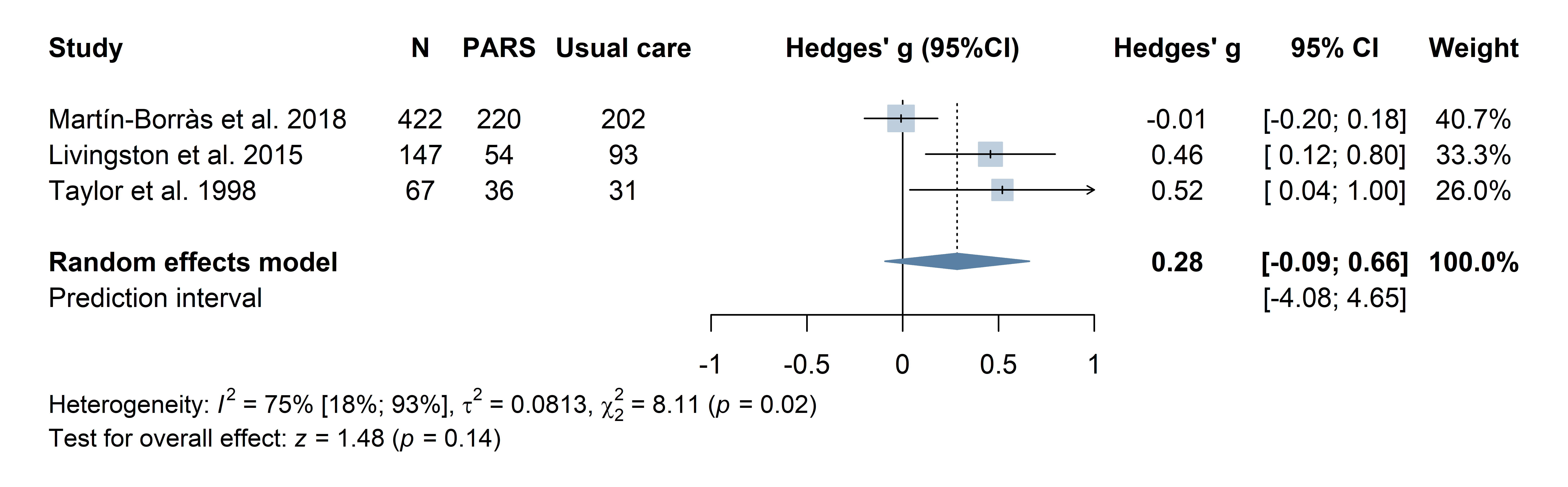


*Influential case*: Omitting Martín-Borràs et al. 2018, g=0.06, 95%CI -0.21 to 0.34, p=0.64

**Moderate PA**

**
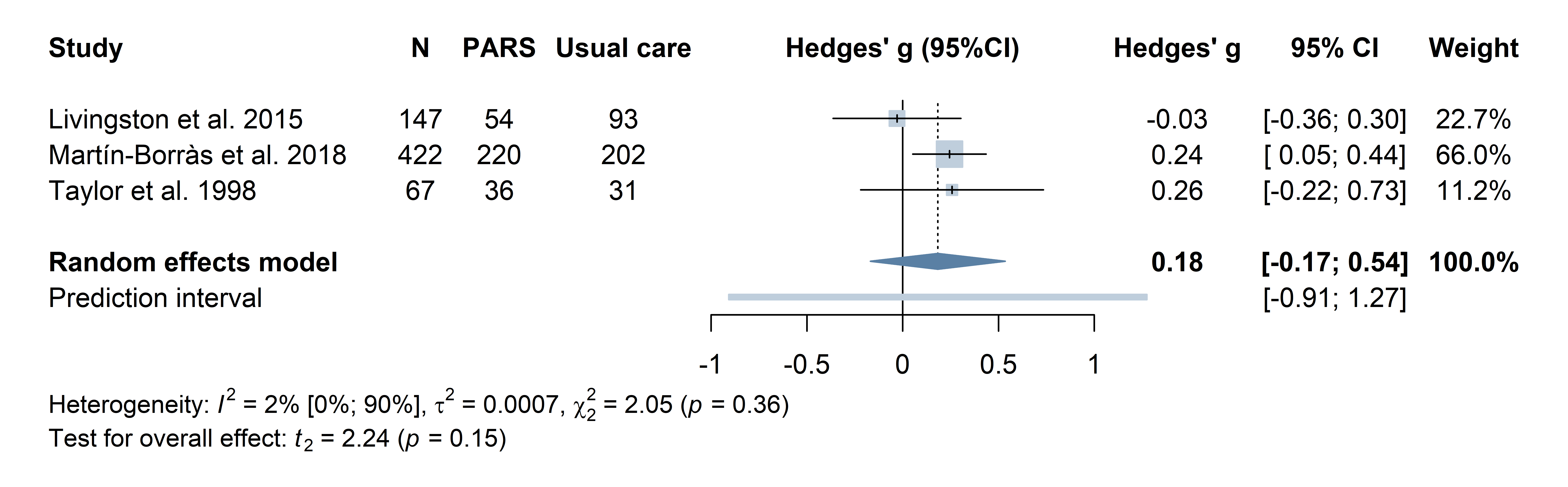
**

**Walking**


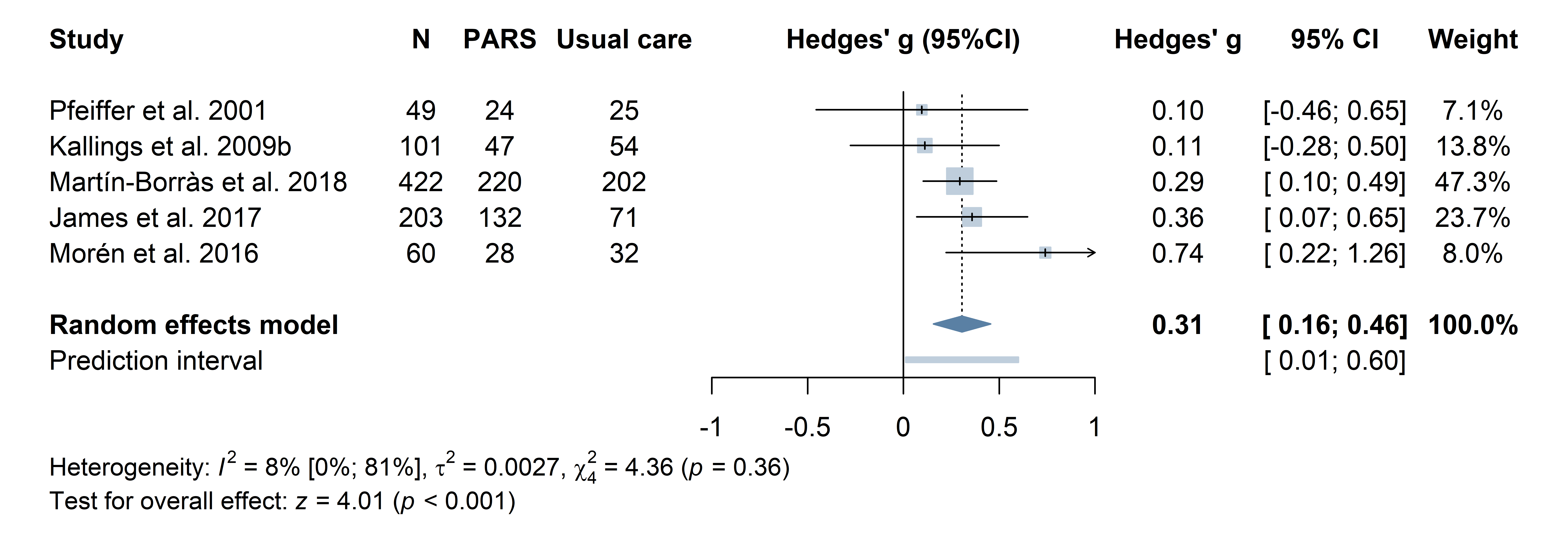


**B) PARS vs physical activity advice**

**Meeting physical activity recommendations**

**
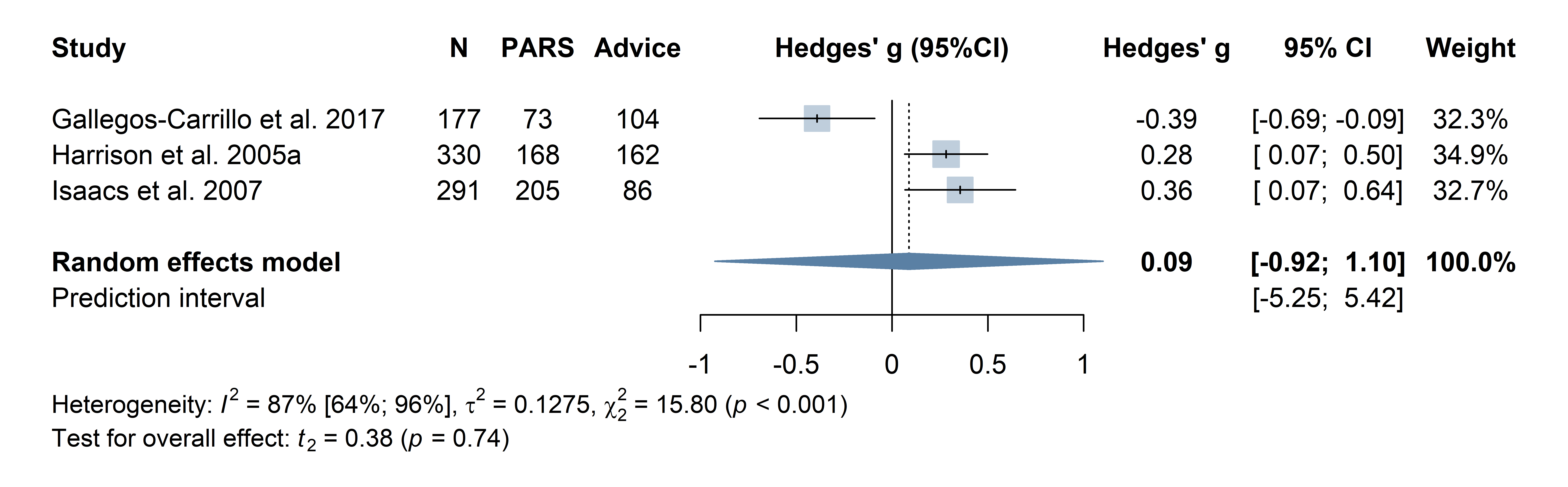
**

**C) Funnel plots**

| 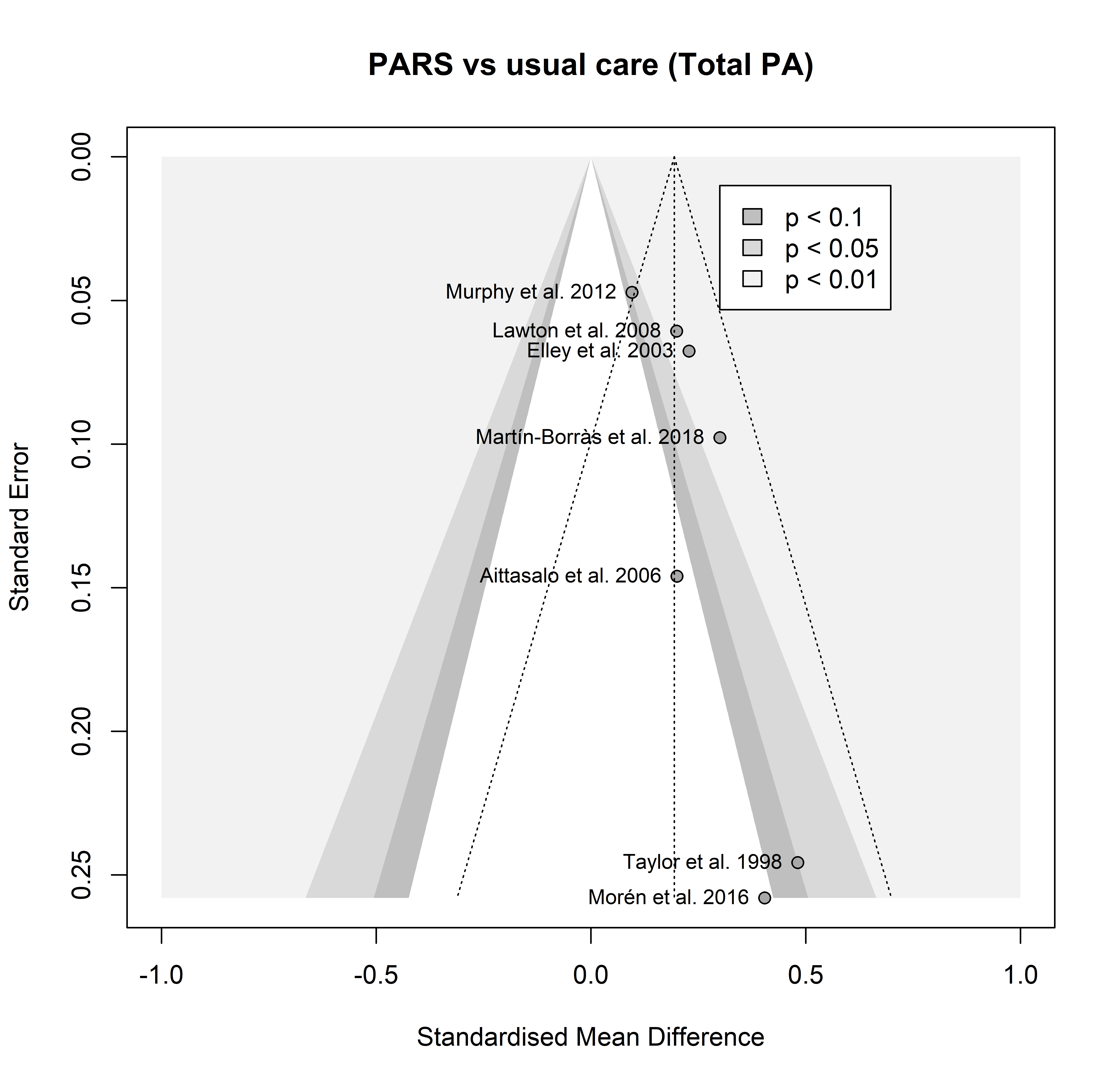 | 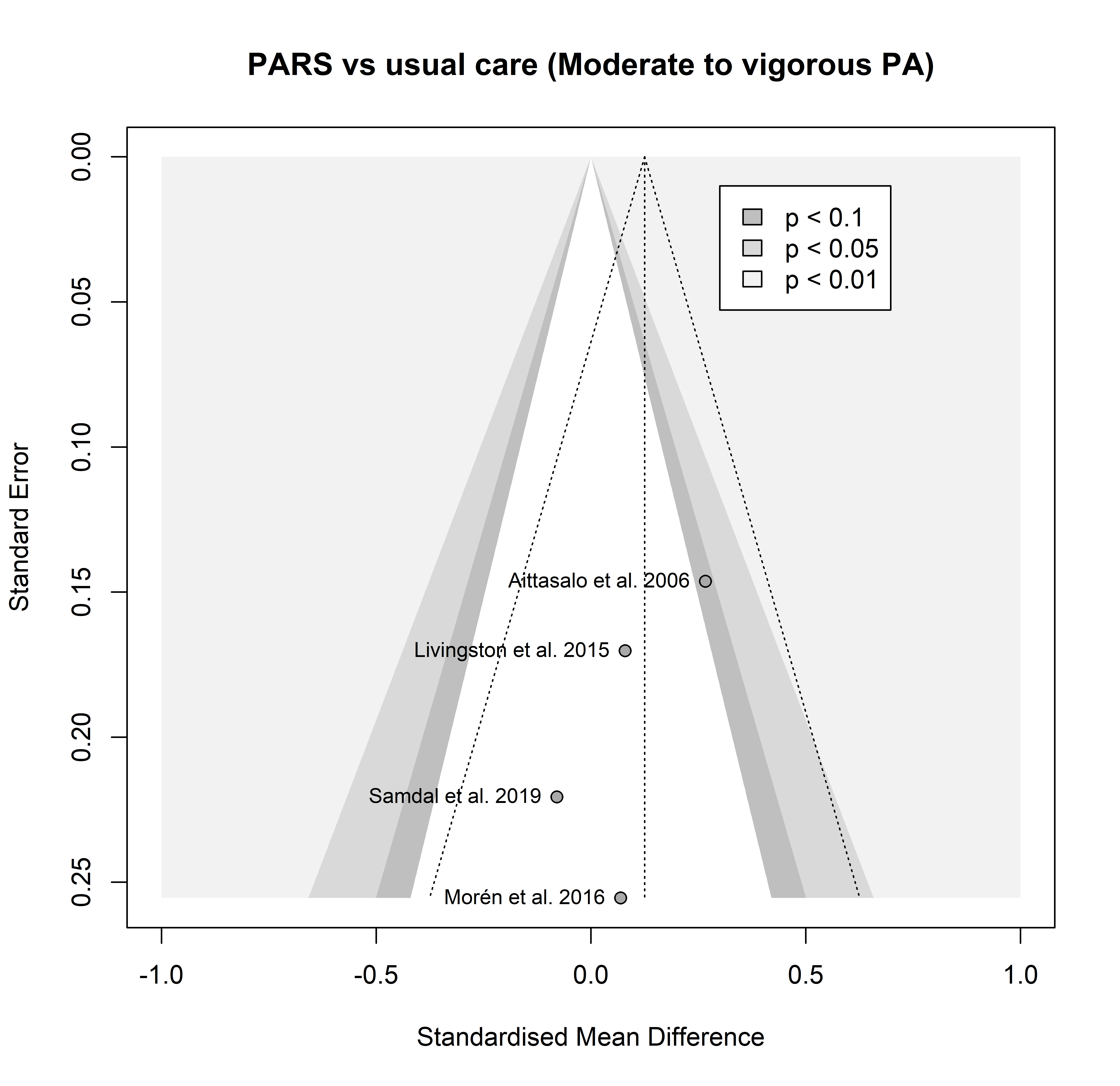 |
| --- | --- |
|  |  |
| 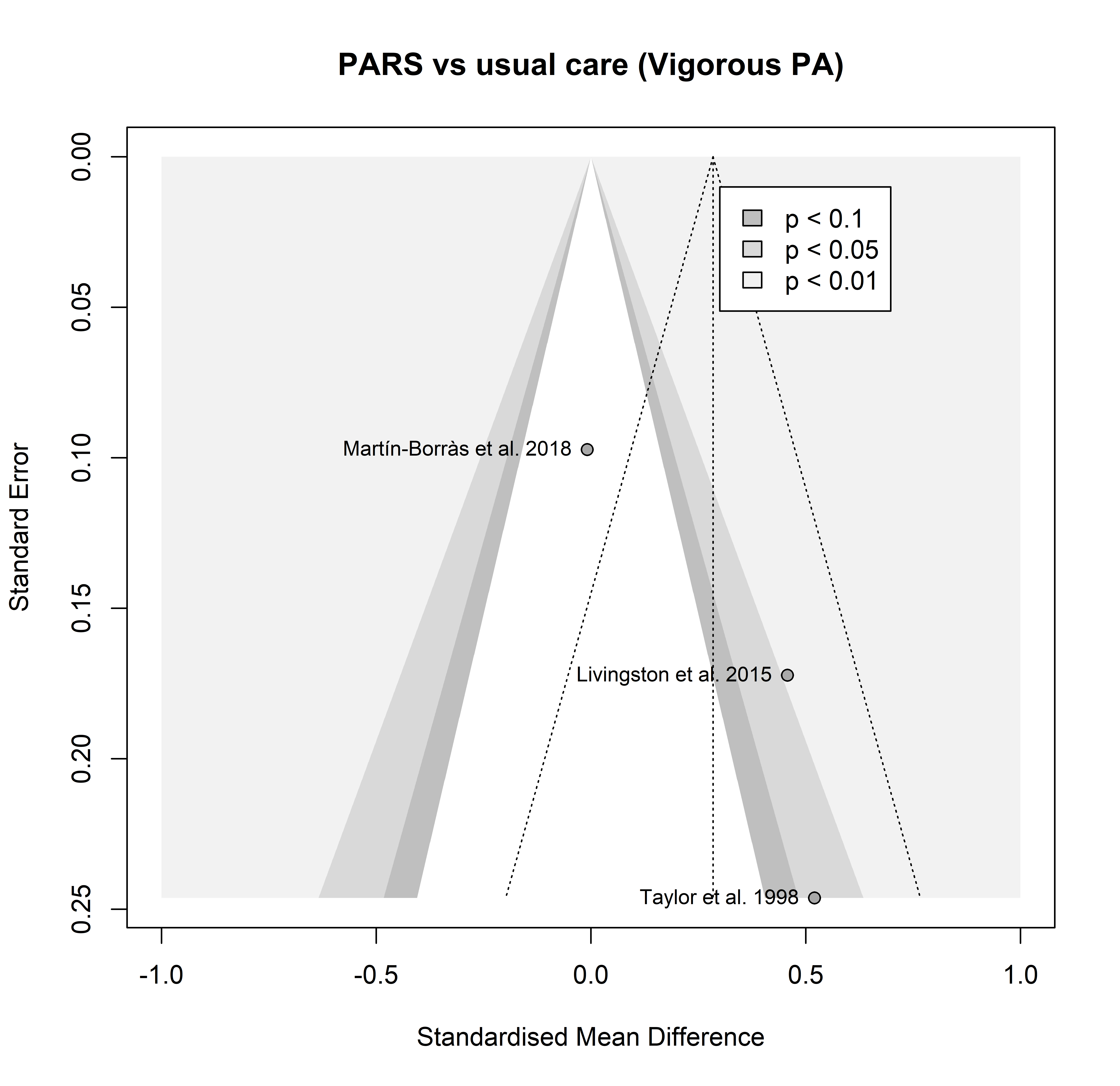 | 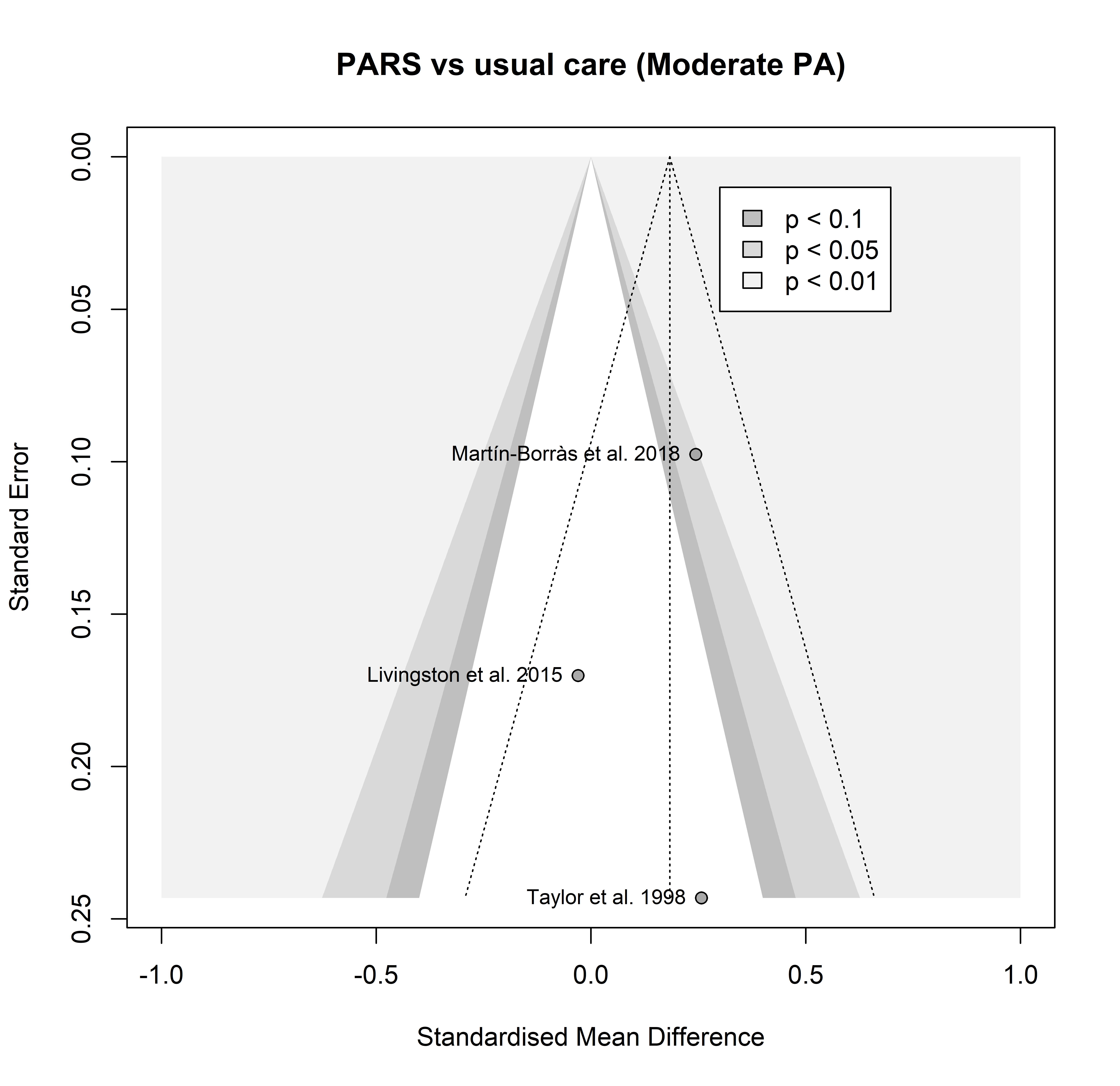 |
| 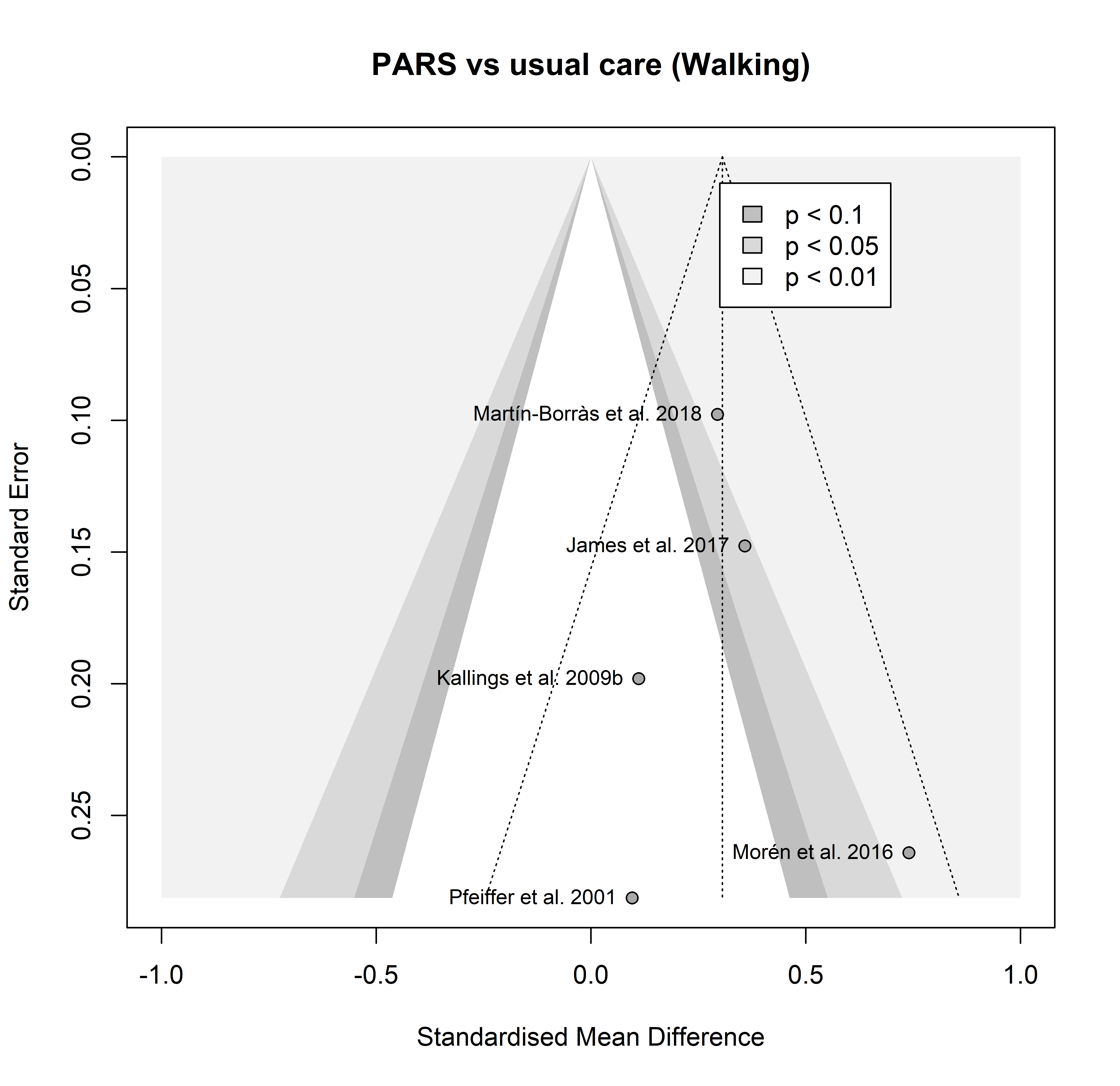 | 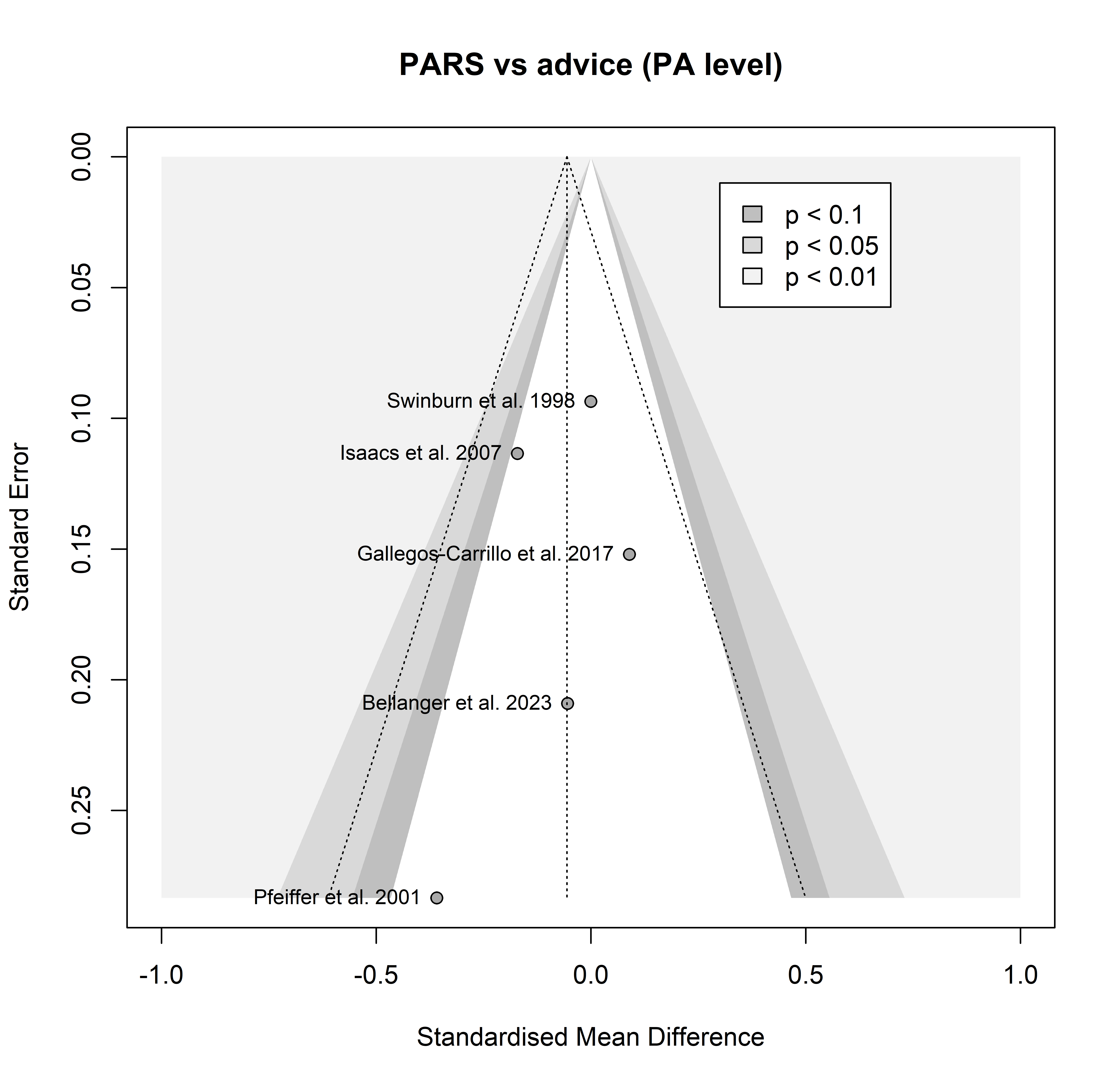 |
