# Supplementary material for: Are physical activity referral scheme components associated with increased physical activity, scheme uptake, and adherence rate? A meta-analysis and meta-regression
Source: Int J Behav Nutr Phys Act. 2024 Aug 2;21:82. doi: 10.1186/s12966-024-01623-5 (PMC11295389; doi:10.1186/s12966-024-01623-5)
Supplement: Supplementary file 10 — Additional file 10. Meta-analysis of randomized trials comparing enhanced with standard PARS for specific physical activity outcomes. [file 12966_2024_1623_MOESM10_ESM.docx]

**Additional file 10.** Meta-analysis of randomized trials comparing enhanced with standard PARS for specific physical activity outcomes

**A) Forest plots**

**Total PA**

**
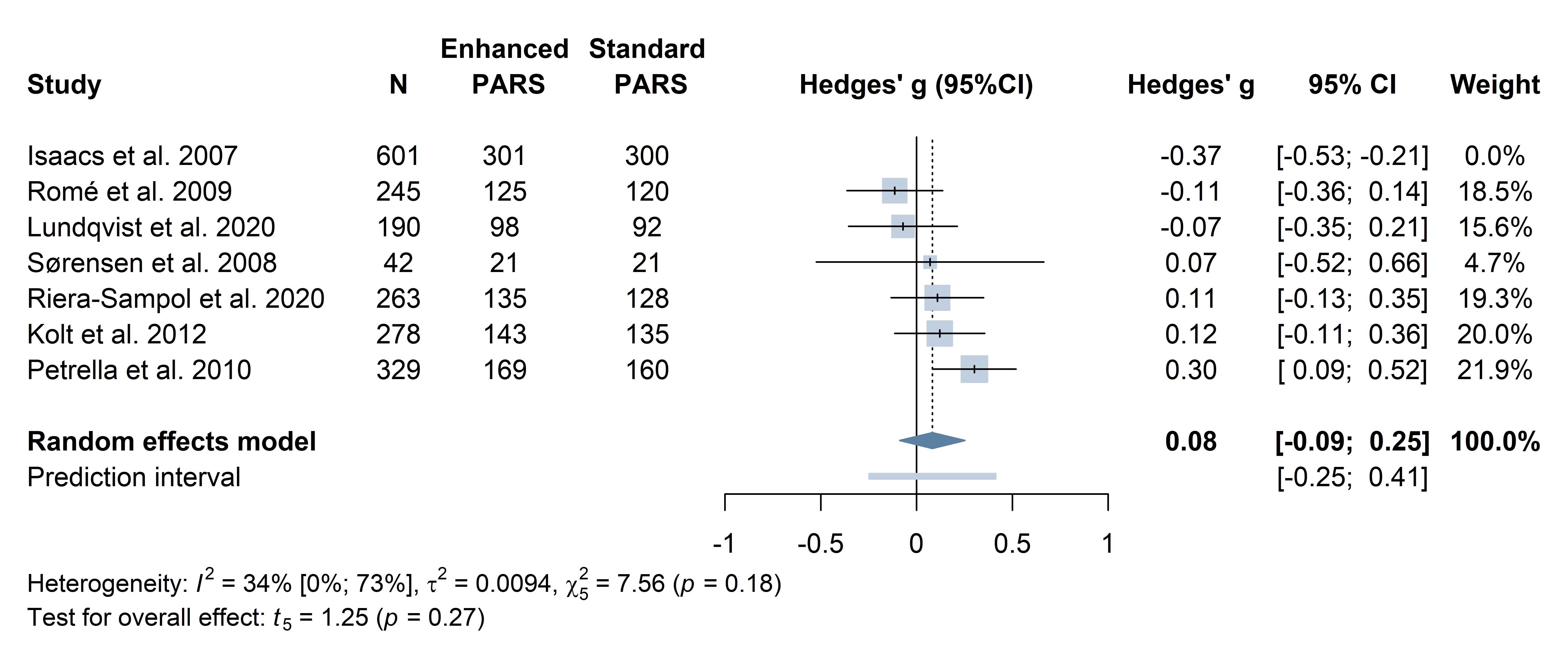
**

Including the outlier Isaacs et al. 2007, g = -0.00 [-0.21; 0.21], p = 0.98, I^2^ = 79.4% [57.8%; 89.9%]

**Meeting PA recommendations**

**
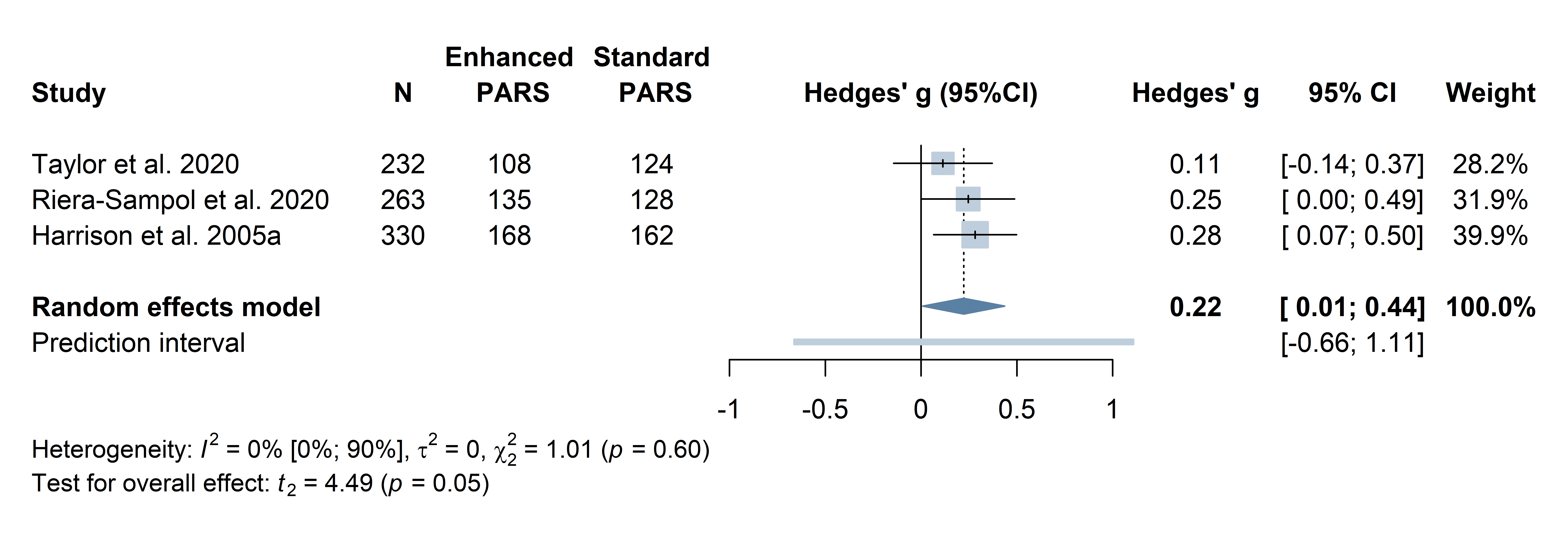
**

**Moderate to vigorous PA**


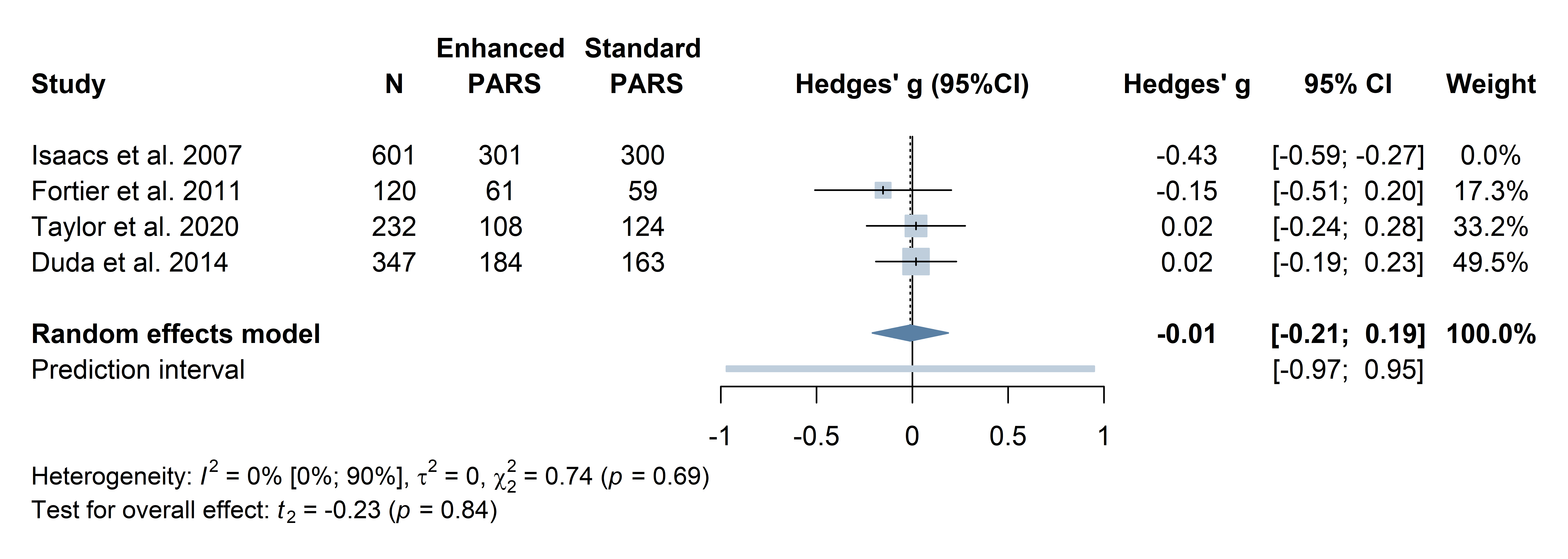


**B) Funnel plots**

| 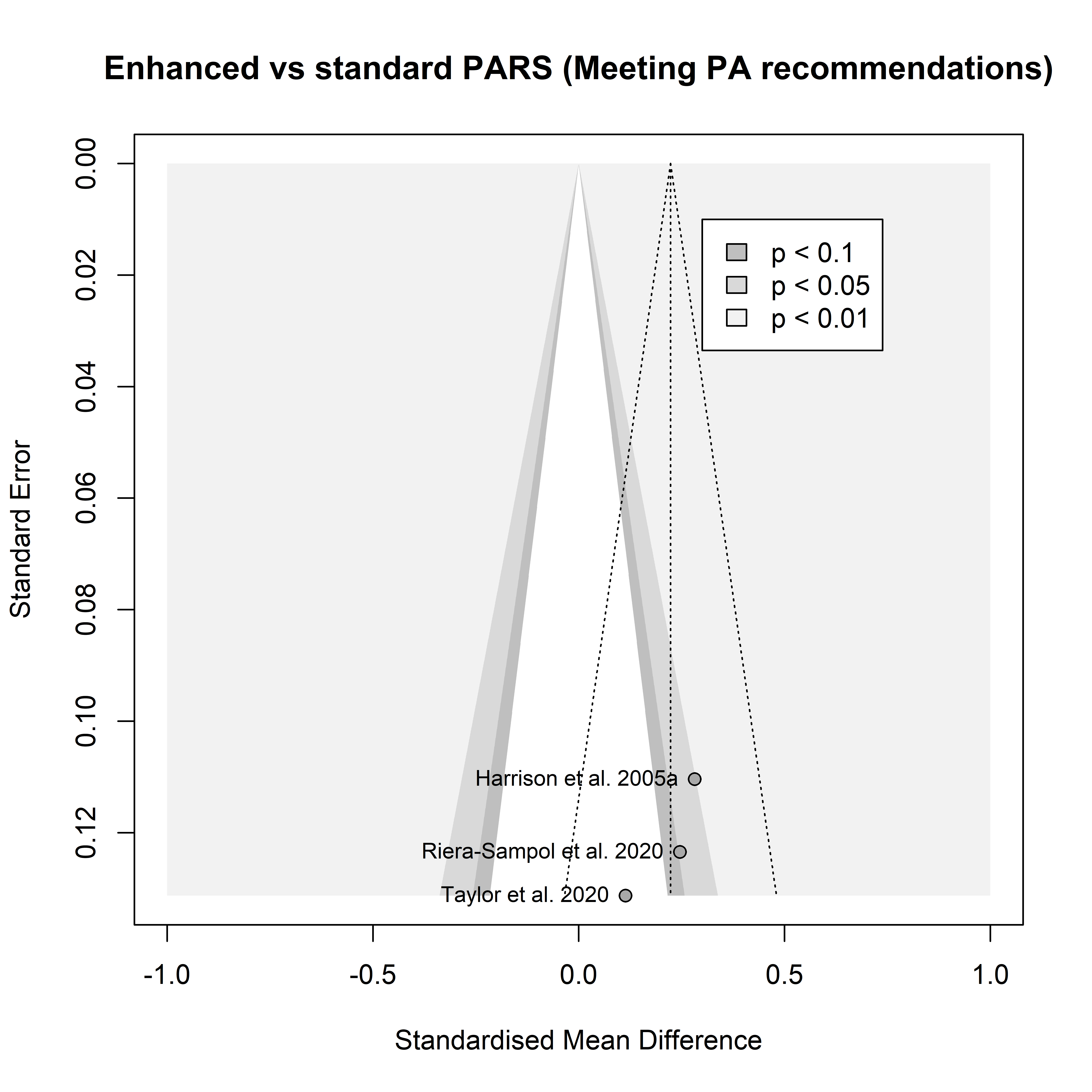 | 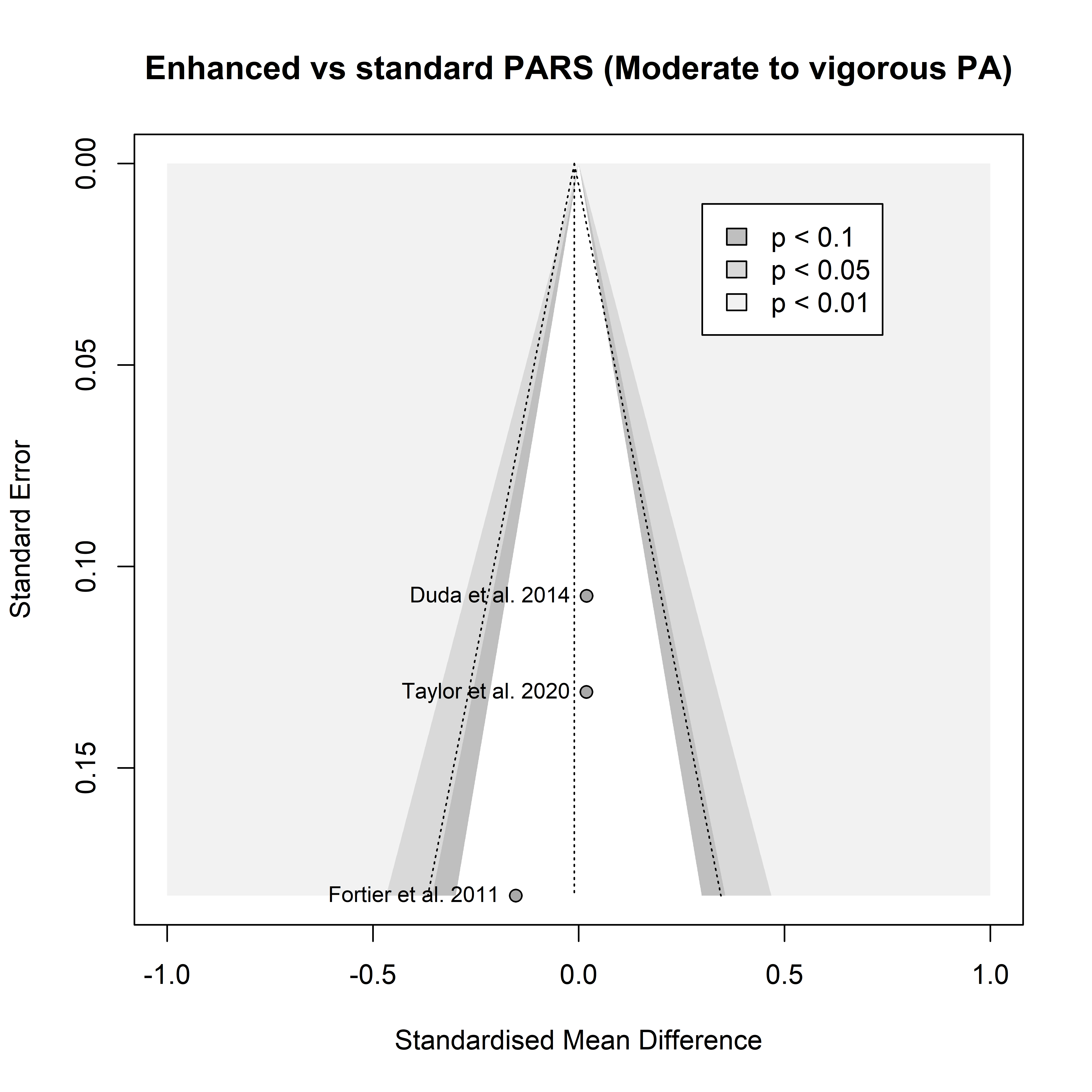 |
| --- | --- |
|  | |
