# Supplementary material for: Are physical activity referral scheme components associated with increased physical activity, scheme uptake, and adherence rate? A meta-analysis and meta-regression
Source: Int J Behav Nutr Phys Act. 2024 Aug 2;21:82. doi: 10.1186/s12966-024-01623-5 (PMC11295389; doi:10.1186/s12966-024-01623-5)
Supplement: Supplementary file 11 — Additional file 11. Meta-analysis of 12 non-experimental studies for physical activity level. [file 12966_2024_1623_MOESM11_ESM.docx]

**Additional file 11.** Meta-analysis of 12 non-experimental studies for physical activity level


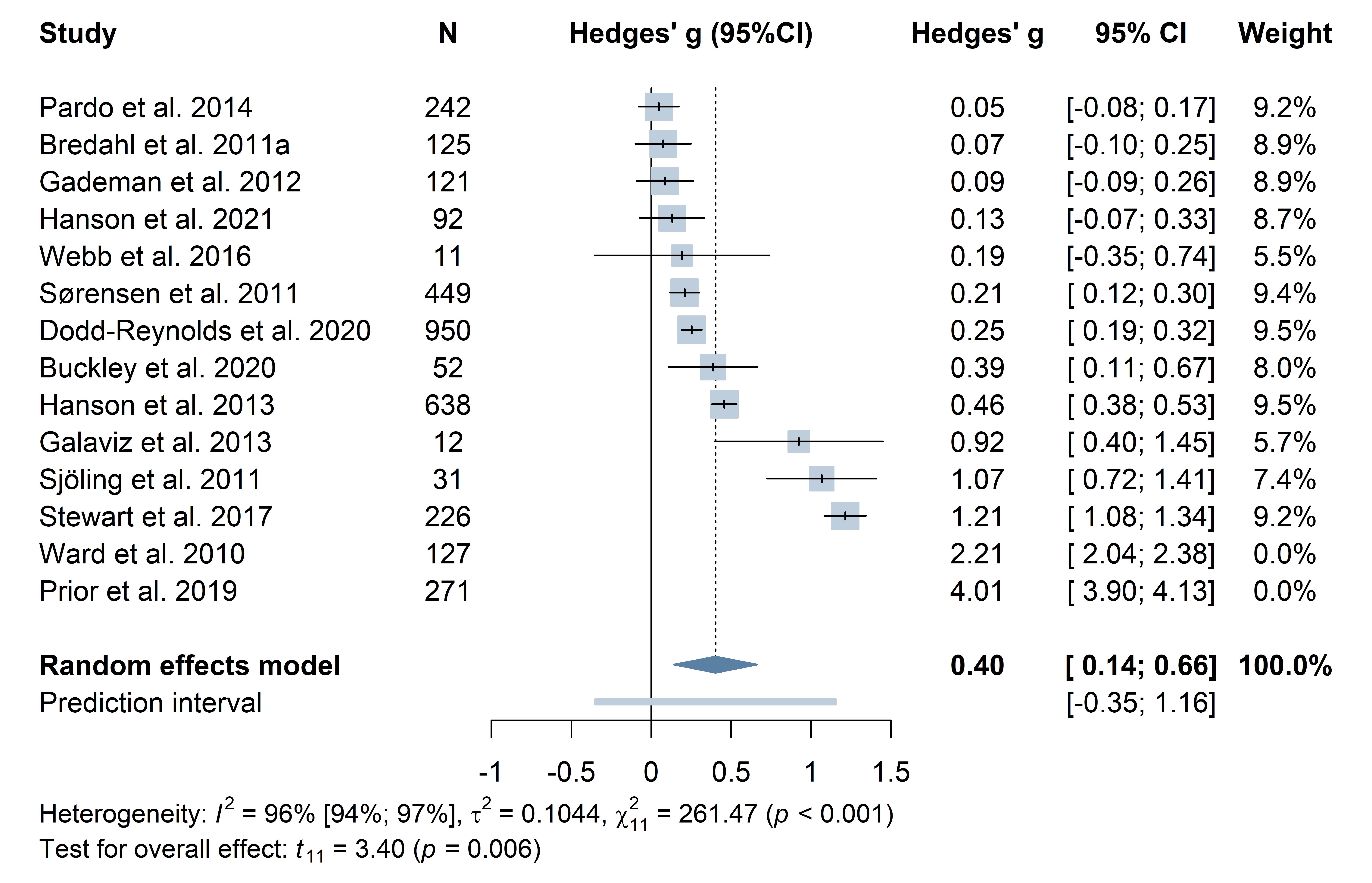


*Outliers not pooled*: Ward et al. 2010, Prior et al. 2019. Including outliers (k=14): g = 0.81, 95%CI: 0.16 to 1.45, p = 0.02;

I^2^ = 99.7%, τ^2^ = 1.38 [0.68; 4.11]


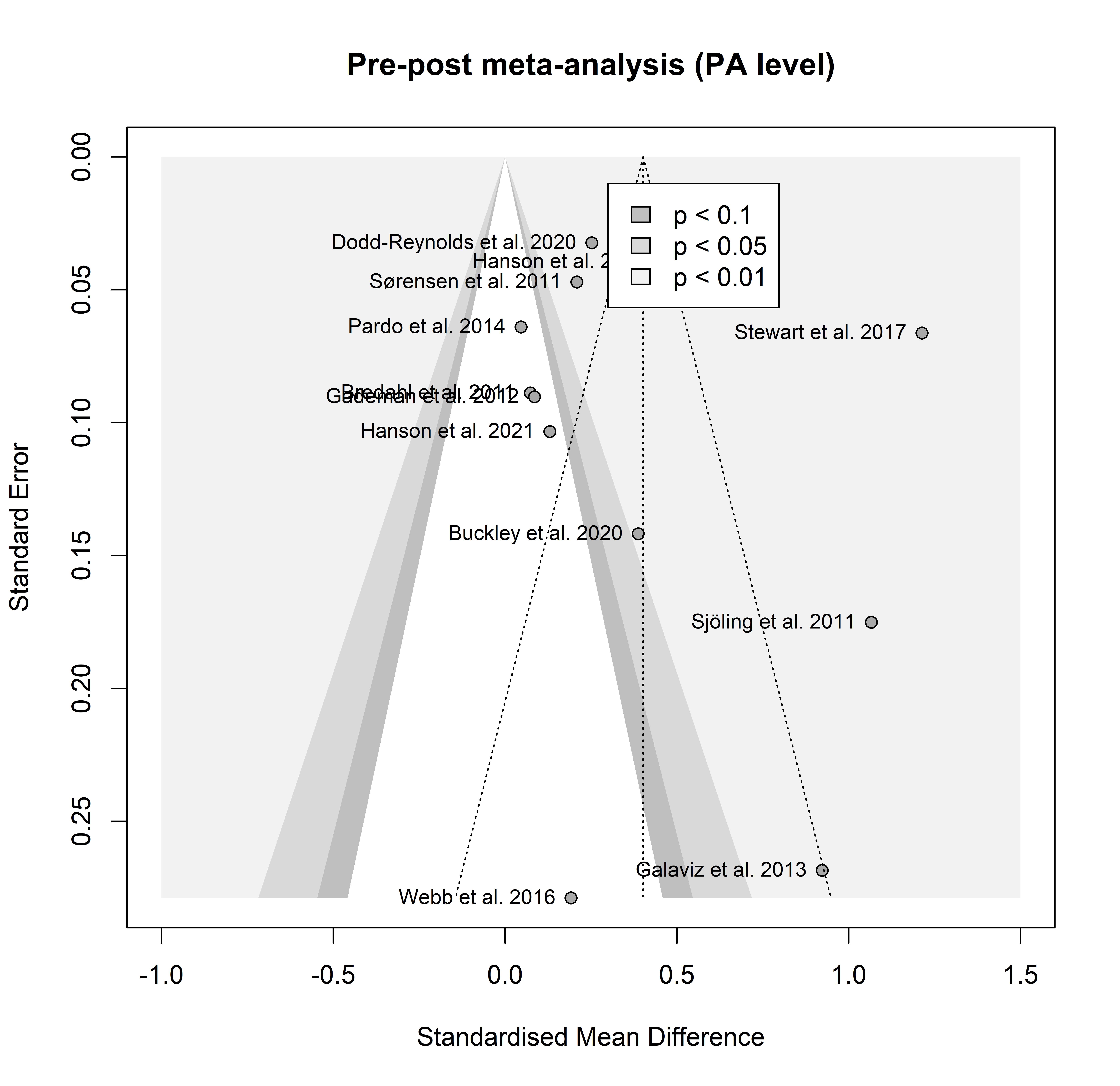


Eggers’ test: intercept ^β0 = 1.176, 95% CI: -4.25 to – 6.6, t = 0.425, p = 0.68
