# Supplementary material for: Are physical activity referral scheme components associated with increased physical activity, scheme uptake, and adherence rate? A meta-analysis and meta-regression
Source: Int J Behav Nutr Phys Act. 2024 Aug 2;21:82. doi: 10.1186/s12966-024-01623-5 (PMC11295389; doi:10.1186/s12966-024-01623-5)
Supplement: Supplementary file 12 — Additional file 12. The relationship between PARS components and effect on physical activity level from 12 pre-post studies. [file 12966_2024_1623_MOESM12_ESM.docx]

**Additional file 12**. The relationship between PARS components and effect on physical activity level from 12 pre-post studies

| Component | PARS with the component | PARS without the component | Regression coefficient (B) | SE | p value | R^2^ (%) | I^2^ (%) |
| --- | --- | --- | --- | --- | --- | --- | --- |
| Individualized content | 7 | 5 | 0.35 | 0.23 | 0.15 | 8.0 | 95.4 |
| Behavior change theory | 5 | 7 | 0.01 | 0.25 | 0.97 | 0.0 | 96.1 |
| Behavior change techniques | 6 | 6 | -0-03 | 0.25 | 0.91 | 0.0 | 96.0 |
| Baseline consultation | 7 | 5 | 0.83 | 0.60 | 0.19 | 0.0 | 99.7 |
| Exit consultation | 7 | 5 | 0.83 | 0.60 | 0.19 | 0.0 | 99.7 |
| Counselling support session(s) | 7 | 5 | 0.74 | 0.61 | 0.25 | 0.0 | 99.7 |
| Exit strategies/  routes | 5 | 7 | -0.95 | 0.58 | 0.13 | 3.52 | 99.6 |

Regression coefficient B: the difference in Hedges’ g between PARS with and without the component used as a predictor variable, SE: standard error of B, p value: significance of B, R^2^: the amount of heterogeneity explained from the component used as a predictor variable, I^2^: residual heterogeneity not accounted for by the component
