# Supplementary material for: Are physical activity referral scheme components associated with increased physical activity, scheme uptake, and adherence rate? A meta-analysis and meta-regression
Source: Int J Behav Nutr Phys Act. 2024 Aug 2;21:82. doi: 10.1186/s12966-024-01623-5 (PMC11295389; doi:10.1186/s12966-024-01623-5)
Supplement: Supplementary file 13 — Additional file 13. Summary of findings table and GRADE evidence profiles. [file 12966_2024_1623_MOESM13_ESM.docx]

**Additional file 13.** Summary of findings table and GRADE evidence profiles

|  | | | | | | |
| --- | --- | --- | --- | --- | --- | --- |
| **Patient or population:** aged inactive individuals ≥ 16 years with or at risk of NCDs  **Setting:** PARS initiated in primary or secondary healthcare, but continued (or not) outside the healthcare setting  **Intervention:** PARS  **Comparison:** usual care, PA advice, enhanced PARS, no comparison | | | | | | |
| Outcomes | **Anticipated absolute effects** | | **Proportion***  (95% CI) | № of participants (studies) | Certainty of the evidence (GRADE) | Comments |
|  | **Hedges’ g**  (95% CI) | |  |  |  |  |
| PA | **0.18** (0.12 to 0.25) | | - | 5046 (11 RCTs) | ⨁⨁⨁⨁ High^a,b,c,d^ | PARS results in a slight increase in PA compared to usual care only. |
| PARS vs PA advice | **-0.06**  (-0.21 to 0.10) | | - | 1082 (5 RCTs) | ⨁⨁◯◯ Low^b,e,f,g^ | There is no evidence to conclude that there is a difference between PARS and PA advice with regard to PA level. |
| Enhanced vs standard PARS | **0.07**  (-0.03 to 0.20) | - | | 2046 (9 RCTs) | ⨁⨁◯◯ Low^g,h,i^ | There is no evidence to conclude that enhanced versions have a higher effect on PA levels than standard PARS.^b^ |
| Uptake rate  (Experimental studies) | - | **0.87**  (0.77 to 0.94) | | 5000  (14 RCTs) | ⨁◯◯◯ Very low^j,k,l,m^ | The proportional meta-analysis was observational in nature, demonstrating the occurrence of uptake among PARS participants. |
| Uptake rate  (Non-experimental studies) | - | **0.68**  (0.51 to 0.83) | | 25’048  (14 studies) | ⨁◯◯◯ Very low^j,k,l,m^ | The proportional meta-analysis was observational in nature, demonstrating the occurrence of uptake among PARS participants. |
| Adherence  (Experimental studies) | - | **0.68**  (0.55 to 0.80) | | 3939  (16 RCTs) | ⨁◯◯◯ Very low^j,k,l,m^ | The proportional meta-analysis was observational in nature, demonstrating the extent to which participants adhered to PARS, without assessing their effect. |
| Adherence  (Non-experimental studies) | - | **0.53**  (0.42 to 0.63) | | 14,605  (18 studies) | ⨁◯◯◯ Very low^j,k,l,m^ | The proportional meta-analysis was observational in nature, demonstrating the extent to which participants adhered to PARS, without assessing their effect. |
| PARS: physical activity referral schemes, PA: physical activity, CI: confidence intervals, * single-arm with no comparison group data  **GRADE Working Group grades of evidence** **High certainty:** we are very confident that the true effect lies close to that of the estimate of the effect. **Moderate certainty:** we are moderately confident in the effect estimate: the true effect is likely to be close to the estimate of the effect, but there is a possibility that it is substantially different. **Low certainty:** our confidence in the effect estimate is limited: the true effect may be substantially different from the estimate of the effect. **Very low certainty:** we have very little confidence in the effect estimate: the true effect is likely to be substantially different from the estimate of effect. | | | | | | |

#### Explanations

a. Risk of bias: Some concerns were mainly due to the lack of a pre-specified analysis protocol which might result in selective reporting. Four out of the 11 studies did not describe allocation concealment. Most of the other domains were at low risk of bias. Three studies rated at high risk of bias contributed with a lower weight to the meta-analysis. When excluding these studies, the results remained the same.

b. Inconsistency: Statistical tests (I², τ²) indicate low between-study variability and there is overlap between confidence intervals.

c. Indirectness: Primary healthcare participants with or at risk of chronic diseases, except on acute transient ischemic attack. All interventions were PARS and they were directly compared to usual care. While PA was measured mainly via questionnaires, four used devices. The time frame of PA measurement ranged from post-scheme to 6 & 9 months (2 studies). However, a longer follow-up is only known to lower the effect size. Thus, we did not downgrade.

d. Impression: Confidence intervals favor PARS and the optimal information size of approximately 380 is reached.

e. Some concerns or at high risk of bias due to outcome measurement and selective reporting.

f. Primary healthcare patients with chronic diseases or at risk. PARS is directly compared to advice only. Consistent follow-up time frame.

g. CI cross the line of no difference.

h. Some concerns or high risk of bias due to lack of allocation concealment, missing data, outcome measurement, or selective reporting.

i. Chronic disease or at-risk population. Subjective, post-scheme measurement of PA.

j. Potential bias in a few studies is unlikely to impact the confidence in the pooled effect.

k. Considerable heterogeneity could not be explained by further subgroup analysis.

l. For the majority of the studies, uptake/adherence rate was not the primary outcome investigated, and for some of them, uptake was not mentioned explicitly as an outcome.

m. CIs do not cross the line of no difference, pooled sample size sufficient.

| GRADE evidence profiles | | | | | | | | | |  |  |  |  |  |
| --- | --- | --- | --- | --- | --- | --- | --- | --- | --- | --- | --- | --- | --- | --- |
| **Certainty assessment** | | | | | | **№ of patients** | | **Effect** |  |  |  |  |  |  |
| **№ of studies** | **Study design** | **Risk of bias** | **Inconsistency** | **Indirectness** | **Imprecision** | **PARS** | **Comparison**  **group** | **Hedges’ g (95% CI)** | **Certainty** |  |  |  |  |  |
| **Physical activity** | | | | | | | | | |  |  |  |  |  |
| **PARS vs usual care** | | | | | | | | | |  |  |  |  |  |
| 11 | randomized trials | not serious^a^ | not serious^b^ | not serious^c^ | not serious^d^ | 2571 | 2475 | **0.18** (0.12 to 0.25)* | ⨁⨁⨁⨁ High |  |  |  |  |  |
| **PARS vs PA advice** | | | | | | | | | |  |  |  |  |  |
| 5 | randomized trials | serious^e^ | not serious^b,f^ | not serious | serious^g^ | 516 | 566 | **-0.06**  (-0.21 to 0.10) | ⨁⨁◯◯ Low |  |  |  |  |  |
| **Enhanced vs standard PARS** | | | | | | | | | |  |  |  |  |  |
| 9 | randomized trials | serious^h^ | not serious | not serious^i^ | serious^g^ | 1044 | 1002 | **0.07**  (-0.03 to 0.20) | ⨁⨁◯◯ Low |  |  |  |  |  |
|  | | | | | | | | | |  |  |  |  |  |
| **Certainty assessment** | | | | | | **№ of patients** | | **Effect** |  |  |  |  |  |  |
| **№ of studies** | **Study design** | **Risk of bias** | **Inconsistency** | **Indirectness** | **Imprecision** | **n** | **Total** | **Proportion (95% CI)** | **Certainty** |  |  |  |  |  |
| **Uptake rate** | | | | | | | | | |  |  |  |  |  |
| 14 | randomized trials | not serious^j^ | very serious^k^ | serious^l^ | not serious^m^ | 3794 | 5000 | **0.87**  (0.77 to 0.94)* | ⨁◯◯◯ Very low |  |  |  |  |  |
| 14 | observational studies | not serious^j^ | very serious^k^ | serious^l^ | not serious^m^ | 16,461 | 25,048 | **0.68**  (0.51 to 0.83)* | ⨁◯◯◯ Very low |  |  |  |  |  |
| **Adherence rate** | | | | | | | | | |  |  |  |  |  |
| 16 | randomized trias | not serious^j^ | very serious^k^ | serious^l^ | not serious^m^ | 2590 | 3939 | **0.68**  (0.55 to 0.80)* | ⨁◯◯◯ Very low |  |  |  |  |  |
| 18 | observational studies | not serious^j^ | very serious^k^ | serious^l^ | not serious^m^ | 6307 | 14,605 | **0.53**  (0.42 to 0.63) | ⨁◯◯◯ Very low |  |  |  |  |  |

* p < 0.05, CI: confidence intervals, PARS: physical activity referral schemes, PA: physical activity

#### Explanations

a. Some concerns were mainly due to the lack of a pre-specified analysis protocol which might result in selective reporting. Four out of the 11 studies did not describe allocation concealment. Most of the other domains were at low risk of bias. Three studies rated at high risk of bias contributed with a lower weight to the meta-analysis. When excluding these studies, the results remained the same.

b. Statistical tests (I², τ²) indicate low between-study variability and there is overlap between CI.

c. Primary healthcare participants with or at risk of chronic diseases, except on acute transient ischemic attack. All interventions were PARS and they were directly compared to usual care. While PA was measured mainly via questionnaires, four used devices. The time frame of PA measurement ranged from post-scheme to 6 & 9 months (2 studies). However, a longer follow-up is only known to lower the effect size. Thus, we did not downgrade.

d. CI favor PARS and the optimal information size of approximately 380 is reached.

e. Some concerns or at high risk of bias due to outcome measurement and selective reporting.

f. Primary healthcare patients with chronic diseases or at risk. PARS is directly compared to advice only. Consistent follow-up time frame.

g. CI cross the line of no difference.

h. Some concerns or high risk of bias due to lack of allocation concealment, missing data, outcome measurement, or selective reporting.

i. Chronic disease or at-risk population. Subjective, post-scheme measurement of PA.

j. Potential bias in a few studies is unlikely to impact the confidence in the pooled effect.

k. Considerable heterogeneity could not be explained by further subgroup analysis.

l. For the majority of the studies, uptake/adherence rate was not the primary outcome investigated, and for some of them, uptake was not mentioned explicitly as an outcome.

m. CI do not cross the line of no difference, pooled sample size sufficient.
